# Epitranscriptome analysis of NAD-capped RNA by spike-in-based normalization and prediction of chronological age

**DOI:** 10.1016/j.isci.2023.108558

**Published:** 2023-11-22

**Authors:** Dean Li, Shuwen Ge, Yandong Liu, Miaomiao Pan, Xueting Wang, Guojing Han, Sili Zou, Rui Liu, Kongyan Niu, Chao Zhao, Nan Liu, Lefeng Qu

**Affiliations:** 1 Interdisciplinary Research Center on Biology and Chemistry, Shanghai Institute of Organic Chemistry, Chinese Academy of Sciences, 100 Hai Ke Road, Pudong, Shanghai 201210, China; 2 University of Chinese Academy of Sciences, Beijing 100049, China; 3 Department of Vascular and Endovascular Surgery, Chang Zheng Hospital, Naval Medical University, Shanghai 200003, China; 4 National Clinical Research Center for Aging and Medicine, Huashan Hospital, School of Basic Medical Sciences, Shanghai Medical College, Fudan University, 131 Dong An Road, Shanghai 200032, China; 5 Metalife Biotechnology, 1000 Zhen Chen Road, Baoshan, Shanghai 200444, China; 6 Singlera Genomics, 500 Fu Rong Hua Road, Pudong, Shanghai 201204, China; 7 Shanghai Key Laboratory of Aging Studies, 100 Hai Ke Road, Pudong, Shanghai 201210, China

**Keywords:** Computational bioinformatics, Sequence analysis, Transcriptomics, Methodology in biological sciences

## Abstract

Nicotinamide adenine dinucleotide (NAD) can be used as an initiating nucleotide in RNA transcription to produce NAD-capped RNA (NAD-RNA). RNA modification by NAD that links metabolite with expressed transcript is a poorly studied epitranscriptomic modification. Current NAD-RNA profiling methods involve multi-steps of chemo-enzymatic labeling and affinity-based enrichment, thus presenting a critical analytical challenge to remove unwanted variations, particularly batch effects. Here, we propose a computational framework, enONE, to remove unwanted variations. We demonstrate that designed spike-in RNA, together with modular normalization procedures and evaluation metrics, can mitigate technical noise, empowering quantitative and comparative assessment of NAD-RNA across different datasets. Using enONE and a human aging cohort, we reveal age-associated features of NAD-capping and further develop an accurate RNA-based aging clock that combines signatures from both transcriptome and NAD-modified epitranscriptome. enONE facilitates the discovery of NAD-RNA responsive to physiological changes, laying an important foundation for functional investigations into this modification.

## Highlights

• enONE, a computational framework of NAD-RNA, reduces technical noise using spike-in
• Human peripheral blood cells contain NAD-RNA and tend to increase with age
• RNA-based aging clock, integrating gene expression with NAD-RNA, can predict age
• NAD-capped RNA from circulating blood can be developed as potential biomarkers

## Graphical abstract

**Figure:**
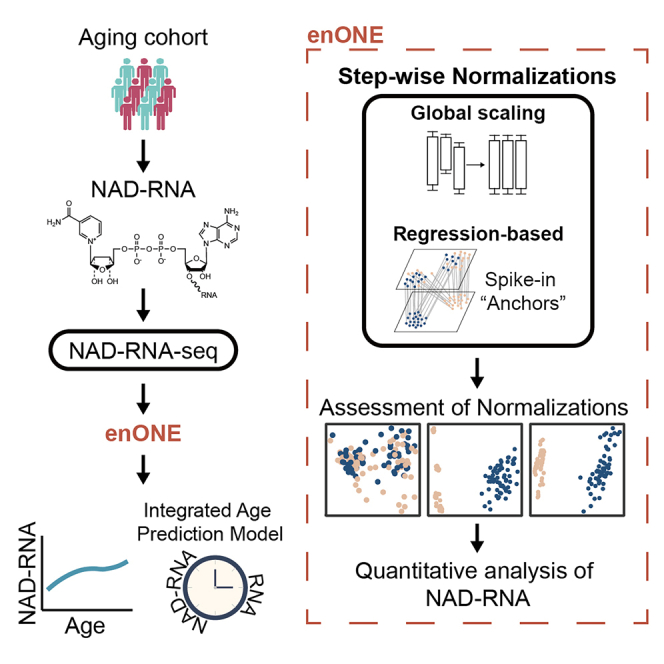

## Introduction

Nicotinamide adenine dinucleotide (NAD), an adenine nucleotide containing metabolite, can be incorporated into the RNA 5′-terminus to result in NAD-capped RNA (NAD-RNA),^1^^,^^2^ which is different from eukaryotic canonical cap structure formed by 7-methylguanosine (m^7^G) via a 5′-to-5′-triphosphate bridge (m^7^G-RNA).^3^^,^^4^ It has been estimated that NAD-capped forms constitute approximately 0.6% and 1.3% of the transcripts expressed in the mouse liver and kidney, respectively.^5^ To capture such low-level capping events, the recently developed NAD-RNA identification methods involve multi-steps of chemo-enzymatic reaction, followed by affinity-based enrichment.^6^^,^^7^^,^^8^ As a consequence, the resulting high-throughput sequencing data can be hampered by the effect of capture procedures and other unwanted variations, e.g., the batch effect. Given these limitations, current computational methods cannot be directly applied to the omic-level quantitative assessment of NAD-capped RNAs.

Proper normalization may serve to remove unwanted variations from enrichment-based sequencing methods. N^6^-methyladenosine (m^6^A), a known epitranscriptomic modification in RNA, has been extensively characterized in virus and eukaryotic organisms by antibody-based immunoprecipitation and sequencing.^9^ Computational tools for m^6^A-seq, e.g., RADAR^10^ and m^6^A-express,^11^ employ a split scaling strategy that calculates scale factors for input and enrichment, respectively, to adjust the variations from enrichment procedures and sequencing depth. However, these analytical methods cannot properly account for the unwanted variation between samples with and without enrichment, thus challenging the identification of enrichment signals. More generally, current analyses of epitranscriptomic data are mostly based on normalization designed for bulk RNA-seq, e.g., scaling-based methods, such as total count (TC), trimmed mean of M values (TMM),^12^ and DESeq.^13^ The implicit assumption underlying scaling-based methods is that all the gene-level counts are proportional to scale factors and that the between-sample variations can be adequately adjusted by scale factors. Unfortunately, this assumption is inevitably violated when affinity-based enrichment selectively amplifies the signal of genes, e.g., m^6^A and NAD capping, which leads to disproportional gene counts between input and enrichment. Another regression-based method, remove unwanted variation (RUV),^14^ regresses gene count measurements on unwanted factors, thus computing corrected expression values from the residuals. The implicit assumption underlying this method is that a set of negative controls, which are not affected by covariates of interest, is available, such as the spike-in from the External RNA Controls Consortium (ERCC). However, the ERCC-based method suffers from discrepancies between endogenous transcripts and spike-in, hindering its usage in omic-level profiling. Limited by current analytical methods, nuisance variations fail to be properly corrected for enrichment-based epitranscriptomic sequencing data, thereby obscuring true biological signals.

NAD, an essential metabolite and redox regent, has been found to decline with age in crucial tissues of mammals including human.^15^ Given the dynamics of NAD and gene expression over the course of adult lifespan, NAD-capped RNA, poised to integrate metabolomics and transcriptomics, may provide novel insights into physiological and perhaps pathological situations. Thus, it is tempting to explore how NAD-modified epitranscriptome is modulated with age. However, analysis of NAD-capped RNAs from heterogeneous populations, particularly those from aging human cohort, presents an additional challenge due to the interrelated variations from individual heterogeneity, impacts of aging, and the technical noises inherent in affinity-based enrichment procedures. Therefore, such complex datasets require a new analytical strategy for disentangling the covariates of interest.

In the present study, we propose enONE computational framework for NAD-capped RNA data analysis by efficiently removing the impact of affinity-based enrichment variations using spike-ins. By identifying genes that capture unwanted variations from exogenous spike-in, referred to as the “anchor set”, enONE can transform data into a shared space, in the presence of extensive technical variations, e.g., batch effect. Based on the spike-in anchor set, enONE integrates global scaling and regression-based normalizations, followed by performance evaluation that selects the local-optimal normalization method to remove unwanted variation. As a computational approach, enONE, combined with NAD-RNA capture and sequencing technology, particularly our recently developed ONE-seq method,^8^ can facilitate quantitative analysis of NAD-RNA profiles. Using human aging cohort, we apply enONE to the identification of NAD-RNAs from human peripheral blood mononuclear cells (PBMCs), revealing critical features and dynamics of NAD modification with age. We further combined transcriptomic and epitranscriptomic features, to develop an RNA-based clock for the prediction of human chronological age.

## Results

### The workflow of enONE

**Figure 1:**
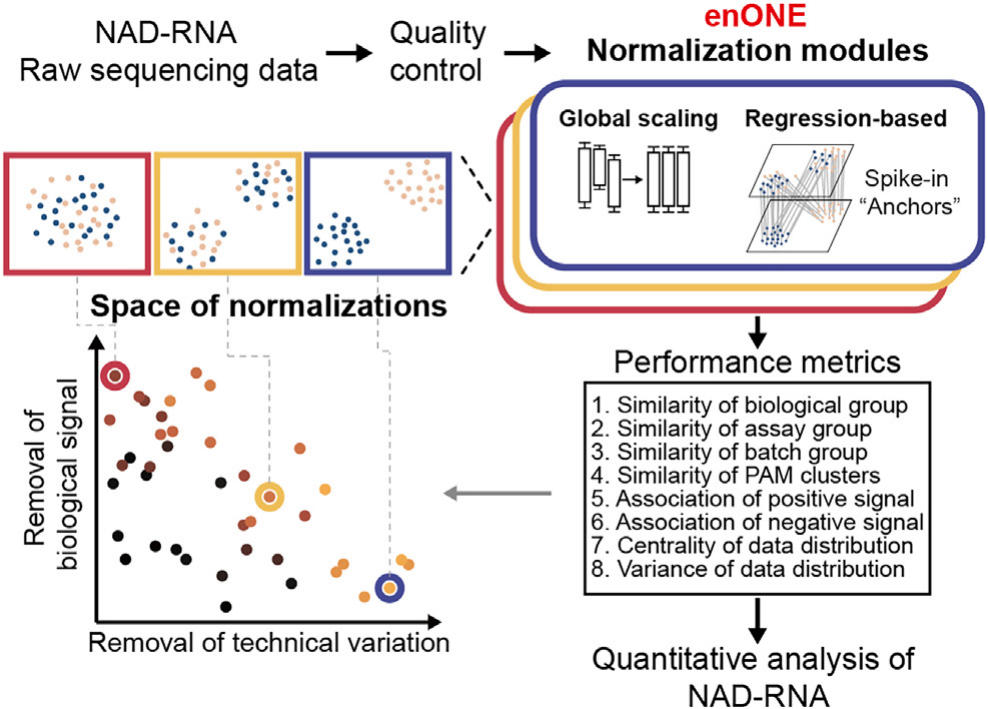
The workflow of enONE enONE starts with quality control to obtain high-quality sequence reads by removing outliers and lowly expressed genes. enONE performs spike-in-based normalization that integrates global scaling and regression-based procedures to generate normalization toolsets. Next, enONE uses eight data-driven metrics to evaluate normalization performance. By exploiting the full space of normalizations, enONE identifies the optimal procedure that maximally removes unwanted variations, while minimally impacting the signals from NAD-RNA-seq data.

We designed a computational framework that implemented and evaluated the performance of various spike-in-based normalization methods for the analysis of NAD-RNA sequencing data. We thereby named our analytical method enONE, for Epitranscriptional NAD-capped RNA analysis by spike-in-based Omic-level Normalization and Evaluation (Figure 1).

enONE initiates with a quality control step, to remove outlier samples and lowly expressed transcripts. Second, a subset of genes from total RNA spike-in, with their expressions presumably not being influenced by the covariates of interest (e.g., enrichment assay or biological condition), are used as the anchor set to estimate unwanted variation (e.g., batch effect). A generalized linear model is then applied to regress the observed read counts from anchor set on the unknown nuisance variables to estimate factors that are subsequently used by the normalization tools for the adjustment of unwanted variation. Third, a two-part normalization template is employed to define an ensemble of the normalization procedures: (1) global scaling of read counts to account for between-sample difference in sequencing depth and other parameters of the read count distribution and (2) regression-based adjustment for unwanted variations. For instance, one can apply a robust scaling procedure, such as TMM, followed by unsupervised procedures to estimate hidden unwanted variations and regress them out of the data (e.g., RUV^14^). Fourth, enONE compares different normalization tools to identify sets of top-performing procedures. Specifically, enONE calculates ranks based on eight performance metrics that represent the local-optimal trade-offs toward removing unwanted variation, preserving biological variation of interest, and maintaining minimal technical variability of global expression. Combined, enONE utilizes a data-driven approach to determine appropriate normalization procedures for the quantitative analysis of NAD-modified epitranscriptome.

### Epitranscriptomic profiling of human PBMCs

**Figure 2:**
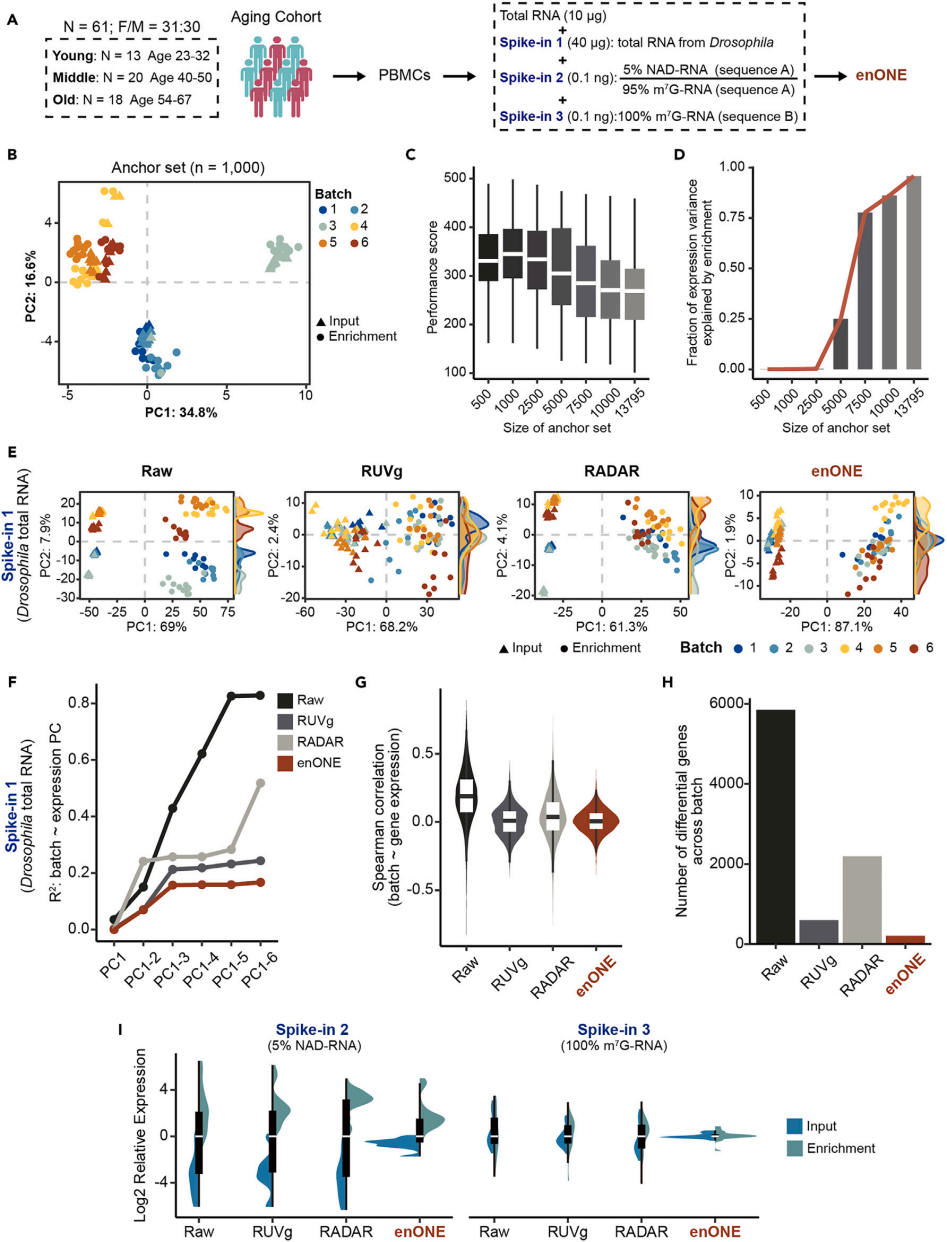
The feasibility of enONE (A) Aging Cohort and experiment outline. A total of 61 participants (female/male = 31:30, age 23–67) were enrolled for NAD-modified epitranscriptome profiling. Total RNAs from PBMCs were mixed with *Drosophila* spike-in RNA, and two synthetic spike-in, one with 5% NAD-capped forms and another with 100% m^7^G-capped forms. The mixture was subjected to NAD-RNA-seq, followed by enONE computational analysis of NAD-RNA profiles (highlighted in red). (B) Scatterplots of first two PCs for the 1,000 least significantly enriched genes (denoted as anchor set) from *Drosophila* spike-in. (C) Boxplot showing the normalization performance based on anchor set of different sizes from *Drosophila* spike-ins. (D) Fraction of the first two expression PCs variance explained values (taken cumulatively) for linear regression model using enrichment variable as the explanatory variable. PCs were computed from the variance stabilizing and transformed matrix of spike-in counts. (E) Scatterplots of first two PCs for *Drosophila* spike-in counts from different procedures. (F) The *R*^*2*^ of linear regression between batch effect and up to the first six PCs (taken cumulatively). (G) Spearman’s correlation coefficients between the raw/normalized counts and batch effect. (H) The number of genes varied among bathes as inferred by ANOVA (p < 0.01). (I) Boxplot showing the relative expression levels of synthetic spike-ins with 5% NAD-caps and that with 100% m^7^G-caps from different methods.

To gain insights of NAD-modified epitranscriptome during aging, we collected human PBMCs from an aging cohort of community subjects comprising of young (N = 23, age: 23–32), middle (N = 20, age: 40–50), and old (N = 18, age: 54–67) individuals for epitranscriptome-wide profiling of NAD-RNAs (Figure 2A), according to the inclusion criteria approved by the Ethics Committee. Clinical characteristics of the participants were evaluated and listed in Table S1.

Since NAD capping is an evolutionary conserved RNA modification,^16^ we reasoned that exogenous total RNA from an invertebrate species can be used as common standards to mitigate unwanted variations from human samples. We deliberately included three types of spike-in RNAs: (1) total RNA from *Drosophila melanogaster*, an invertebrate model organism with well-annotated genome sequence; (2) a synthetic RNA consisting of 5% NAD relative to m^7^G-capped forms, used to determine the capture sensitivity; and 3) a synthetic RNA with 100% m^7^G-capped form, used to determine the capture specificity (Figure 2A). Notably, spike-ins 2 and 3 were synthesized with templates from different sequences. Combined, we subjected total RNAs from human PBMCs, *Drosophila*, spike-in 2, and spike-in 3 to ONE-seq-based capture and sequencing, followed by enONE computational analysis of NAD-RNA profiles.

After quality control, we obtained an average about 49.2 million high-quality and uniquely mapped sequencing reads from each library (Figure S1A). Assessment of datasets corroborated that sequencing saturation has been reached (Figure S1B). Spike-in 2, which contained 5% NAD-capped form, was significantly enriched, whereas no enrichment was found for spike-in 3 made up with 100% m^7^G-RNA (Figure S1C). Above evidence highlighted the sensitivity and specificity of the enrichment experiment, as reflected by the enrichment of NAD-capped, but not m^7^G-capped, transcripts.

### The feasibility of enONE

Since all samples were added with equal amounts of *Drosophila* total RNA as spike-in, its disconcordance, if present, can be used to pinpoint the nuisance technical variation in an epitranscriptome-wide manner, and its concordance, on the other hand, can be used to validate the effect of normalization. To capture unwanted variation, i.e., batch effect, we used a set of genes (n = 1,000) whose expression patterns should be highly reproducible and now become differed among batches as the anchor set (Figure 2B). In addition, we showed that normalization procedures were robust when the enrichment effect accounted for a small fraction of the anchor set variance, e.g., anchor set size ranged from 500 to 2,500 (Figures 2C and 2D). With anchor set from *Drosophila* spike-in RNA, we implemented enONE normalization procedures with five scaling toolsets, including TC, UQ, TMM, DESeq, and PoissonSeq,^17^ as well as three regression-based procedures, namely RUVg, RUVs, and RUVse. By integrating two normalization modules, we generated a total of 96 combinatorial procedures for the current data (Figure S2A). By inspecting the full space of normalization performance metrics, we found that the top-ranked procedure involved DESeq scaling followed by RUVg adjustment for the first 4 factors of unwanted variation (Figures S2B and S2C). Additionally, this integrated procedure showed that the combination of global scaling and regression-based procedures could improve the normalization performance.

To compare the effect of normalization, we applied three different methods—RUVg (k = 4), a regression-based method; RADAR, a scaling-based method; and enONE—on all three types of spike-in RNAs. Compared to other procedures, enONE normalization dramatically mitigated the batch effect of *Drosophila* spike-in in both input and enrichment libraries, while preserving the enrichment signals (Figure 2E). In *Drosophila* spike-in, linear regression analysis between the first six PCs cumulatively and batch effect showed that batch effect explained over 83% of the variations in raw count data. This variation was reduced to approximately 24% and 52% with the RUVg and RADAR methods, respectively. Significantly, enONE normalization further mitigated the variance explained by batch effect to approximately 17% (Figure 2F). Analysis of correlation between individual gene expression values from *Drosophila* spike-in and batch variation revealed a large proportion of genes showing strong correlations with batch effect in raw, RUVg, and RADAR datasets, whereas this correlation was dramatically diminished by enONE (Figure 2G). ANOVA was performed on *Drosophila* spike-in datasets from different batches to evaluate the impact of batch effect on gene expression measurements. Ideally, there should be minimal difference in gene expression across batches; however, over 5,800 genes were found to be differentially expressed in the raw dataset, reflecting strong batch effect. enONE was able to abolish the batch effect by reducing 96% of such differentially expressed genes, whereas RUVg and RADAR could reduce 90% and 62% of these genes, respectively (Figure 2H). Since synthetic spike-ins were not used to estimate unwanted variation factors, their concordance could serve as an independent validation of normalization performance. The boxplots of raw relative log-expression showed large differences among synthetic spike-ins with equal input amounts (Figure 2I). Compared to RUVg and RADAR, enONE clearly reduced the overall variations of synthetic spike-ins as illustrated by the lower interquartile range. Additionally, enONE normalization preserved the distribution differences between input and enrichment samples of NAD-capped, but not m^7^G-capped, spike-ins, indicating that enONE could retain enrichment signals from transcripts with NAD-cap, but not m^7^G-cap (Figure 2I).

**Figure 3:**
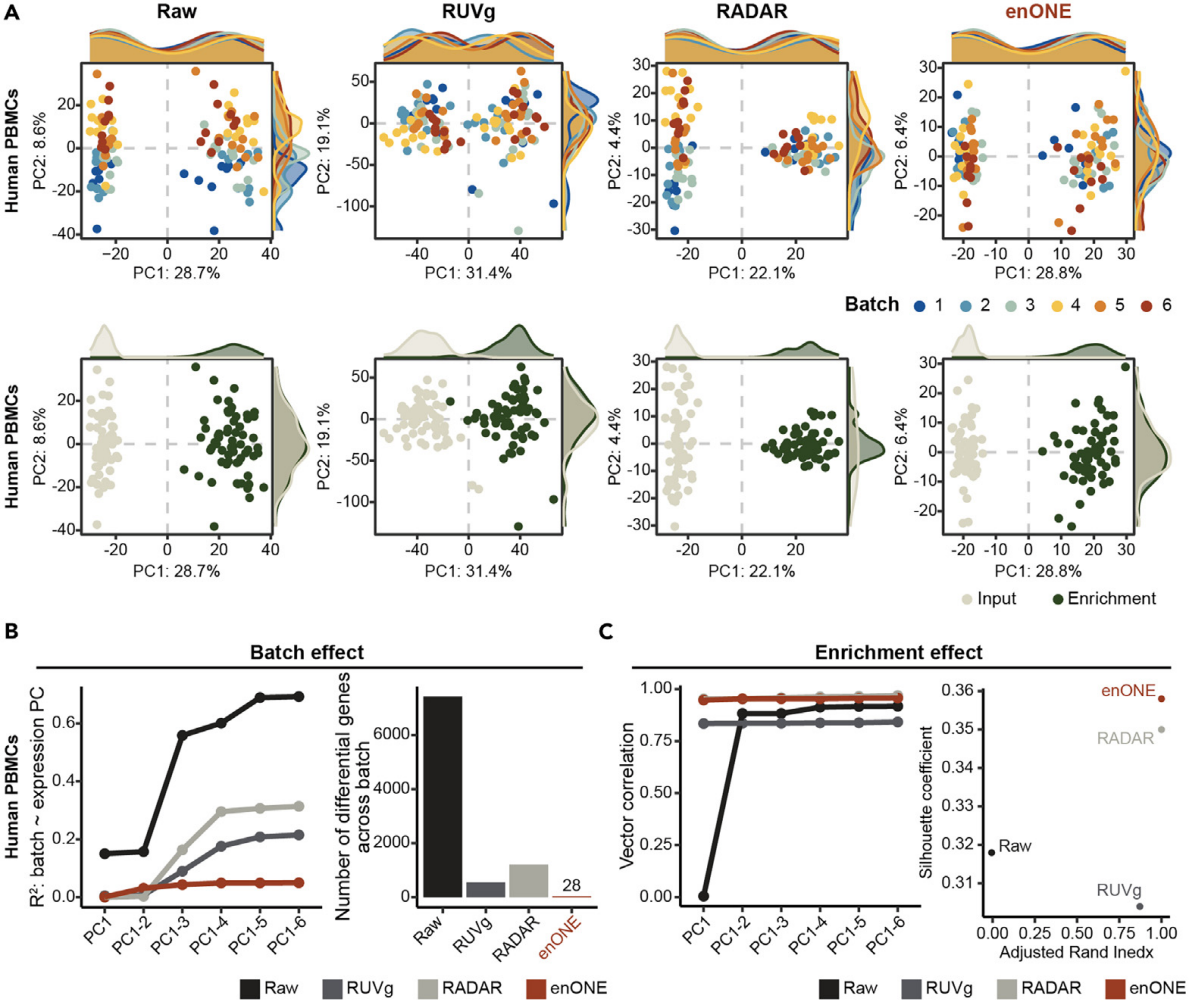
Performance assessment of different normalizations on human PBMCs dataset (A) Top row: scatterplots of first two PCs for human PBMCs counts from different procedures colored by batch. Bottom row: same as the top row colored by the enrichment assay. (B) Left: the *R*^*2*^ of linear regression between batch effect and up to the first six PCs (taken cumulatively). Right: the number of genes varied among bathes as inferred by ANOVA (p < 0.01). (C) Left: line chart showing the vector correlation coefficient between enrichment effect and the first six PCs (taken cumulatively). Right: scatterplot showing silhouette coefficient and adjusted Rand index (ARI) for measuring the separation of enrichment groups.

We next compared the performance of enONE with other normalization approaches using human PBMCs dataset. Although the RUVg and RADAR reduced the variations, both methods had limitations; specifically, RUVg failed to preserve the enrichment signal and RADAR overcorrected the variation among enrichment samples (Figure 3A). Principal component analysis plots illustrated that enONE improved upon the RUVg and RADAR normalizations in removing the variation of batch effect and preserving the enrichment signals (Figure 3A). To examine batch effects in raw and normalized data, we performed linear regression analysis and ANOVA, which showed that enONE exhibited improved performance than RUVg and RADAR (Figure 3B). Furthermore, we evaluated the preservation of covariate of interests, i.e., enrichment effect. By performing vector correlation analysis between the first six cumulative PCs and the enrichment covariate, we noted that over 97% of the enrichment variations were well preserved by enONE (Figure 3B). Besides, the silhouette coefficient and adjusted Rand index analysis was used to assess the separation of input and enrichment samples. Consistently, this result showed that enONE outperformed other procedures in retaining the enrichment signals (Figure 3C). Together, these results demonstrate the capacity of enONE in removing unwanted variation while retaining the covariates of interest.

### Characterization of NAD-RNAs from human PBMCs

**Figure 4:**
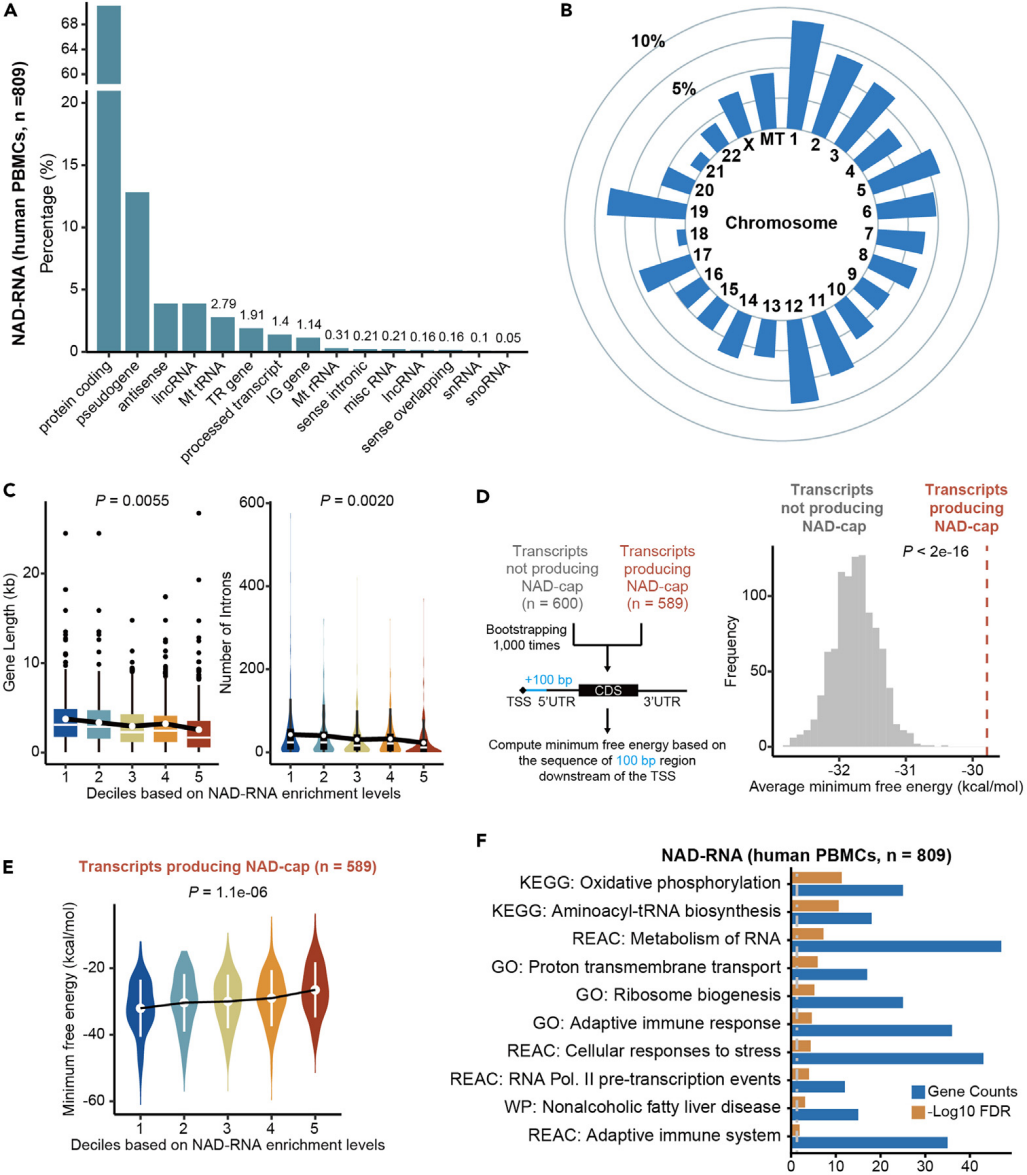
enONE facilitates NAD-capped RNAs identification (A) Barplot showing the gene types of identified NAD-RNAs (n = 809) from human PBMCs. (B) Circular bar plot showing the chromosomes distribution of identified NAD-RNAs. (C) From five deciles based on enrichment, genes with short length and with fewer introns tended to have increased modification of NAD. p values were obtained with ANOVA. (D) Left: for transcripts with 5′ untranslated region (UTR), the 100 bp region downstream of transcription start site (TSS) is used to compute the MFE. Using bootstrapping, the background distribution of MFE was generated based on the transcripts that did not produce NAD-cap. Right: transcripts that produced NAD-cap have predicted structures with higher MFE levels than those did not. p value was estimated with bootstrapping (n = 1,000). (E) From five deciles based on enrichment, genes with higher MFE levels tend to have increased NAD capping. p value was obtained with ANOVA. (F) Pathway analysis reveals the biological processes of NAD-capped RNAs that are mainly involved in RNA metabolism, transcription, translation, and immune system. Gray dashed line in the bar plot indicates the 0.05 FDR cutoff.

We proceeded to set 2-fold enrichment of read counts as the cutoff, which led us to identify a total of 809 NAD-RNAs from human PBMCs (Figure 4A and Tables S2, S3, and S4). We then characterized these newly identified NAD-RNAs. In human PBMCs, NAD capping mostly occurred on protein-encoding genes, but also extended to pseudogenes and non-coding RNAs, including lincRNA, snRNA, snoRNA, and miscRNA (Figure 4A). NAD-RNAs were shown to be derived from genes localized on autosomes, X chromosomes, and the mitochondrion genome, but not from the Y chromosome (Figure 4B). By dividing NAD-RNAs into 5 deciles based on enrichment, we observed that genes with short length and fewer introns tended to have increased modification of NAD (Figure 4C), a pattern consistent with our recent study in mouse livers.^8^ Since previous studies have shown that RNA capping events can be influenced by the proximal RNA secondary structure,^18^ we examined the sequence feature using the 100 bp region downstream of the transcription start site. By leveraging the minimum free energy (MFE),^19^ we observed that transcripts with NAD-cap tended to adopt predicted structures with higher MFE level than those with m^7^G-cap (Figure 4D). Furthermore, transcripts with increased MFE level positively correlated with the extent of NAD modification (Figure 4E), suggesting that RNA transcripts with a stable cap-proximal structure might promote NAD capping. To inspect NAD modification of genes associated with biological functions, we performed pathway enrichment analysis, which revealed that NAD-RNAs from human PBMCs were mainly involved in RNA metabolism, translation, transcription, energy metabolism, and immune system (Figure 4F and Table S5).

### Age alters NAD-modified epitranscriptome

**Figure 5:**
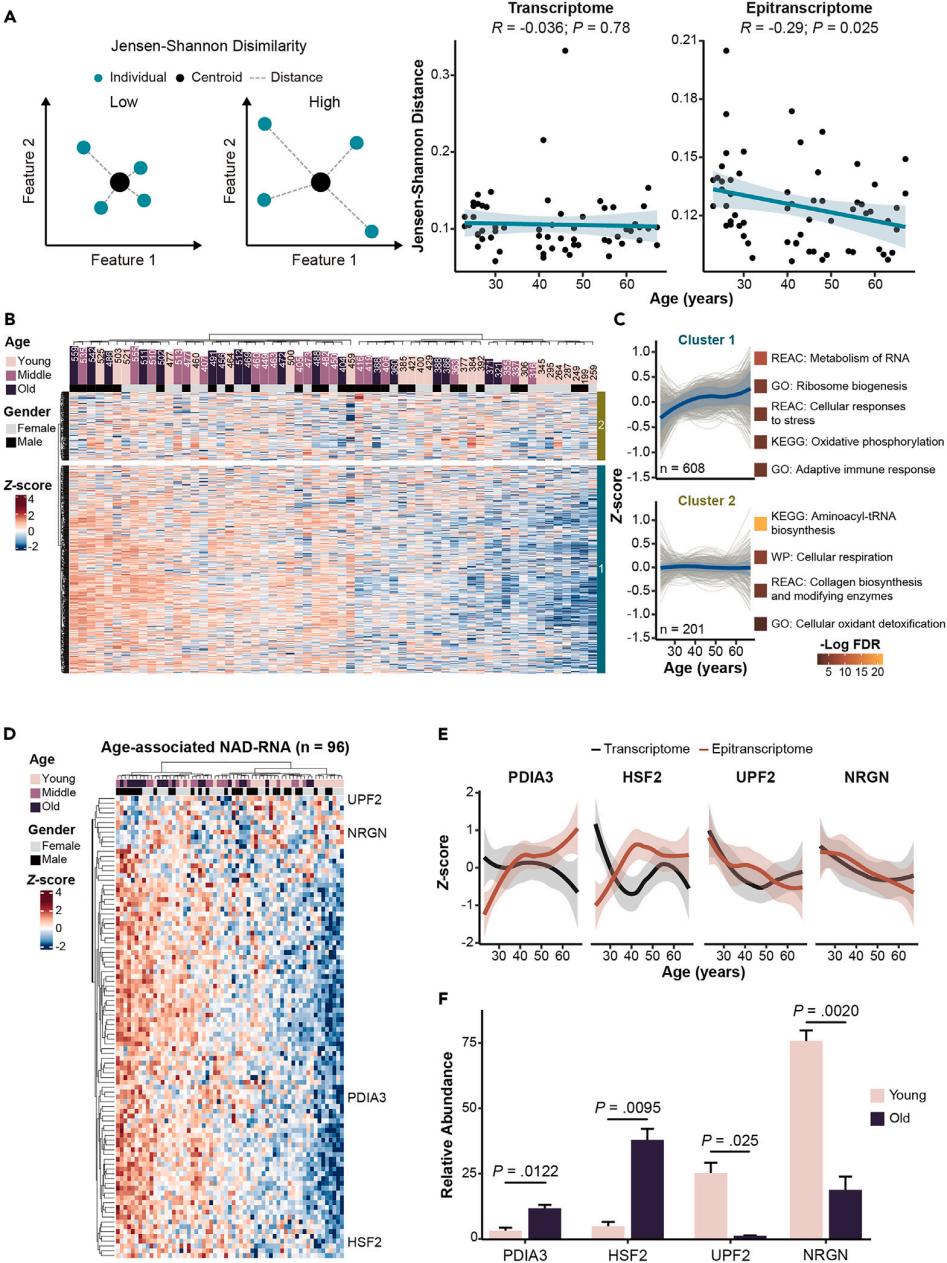
enONE reveals dynamics of NAD-capped RNAs during aging (A) Left: diagram illustrating the Jensen-Shannon distance. The Jensen-Shannon distance was used to quantify the dissimilarity between an individual’s profile and their corresponding reference profile, represented by the centroid. Right: scatterplot showing the relationship between age and the Jensen-Shannon distance from transcriptomic or epitranscriptomic profiles, respectively. The *R* represented the Pearson’s correlation coefficients and the p values were obtained from two-sided Pearson correlation test. (B) Heatmap showing epitranscriptomic profiles from individuals across age groups (N = 61). Top bar represented the enrichment signals (fold-change ≥2) of each sample. (C) Unsupervised hierarchical clustering was used to group NAD-RNAs with similar trajectory. Two major clusters were identified, and the top five non-redundant pathways were listed. The solid line and shaded region represent the smoothed trajectory and its 95% confidence intervals, respectively. (D) Heatmap showing a set of NAD-RNAs (n = 96) that highly associated with age. (E) *Z*-transformed, smoothed trajectories of NAD capping levels (in red) and expression levels (in black) of selected genes were shown. The solid line and shaded region represent the smoothed trajectory and its 95% confidence intervals, respectively. (F) Assessment of gene-specific NAD capping by qRT-PCR. Selected NAD-RNAs, including age-increased NAD-RNAs (PDIA3 and HSF2), as well as age-decreased NAD-RNAs (UPF2 and NRGN), were examined. Data were shown in mean ± s.e.m. (one-sided Student’s *t* test).

To assess the heterogeneity of individual subjects, we utilized the Jensen-Shannon distance (JSD) metric to evaluate the variation in transcriptomic or epitranscriptomic distributions relative to their reference distribution, i.e., centroid.^20^^,^^21^ A JSD value of 0 indicates an equal distribution between the reference and sample, while a JSD of 1 represents the greatest difference between the reference and sample. The transcriptome-derived JSD metrics had an average value of 0.10 and exhibited no significant correlation with ages (Pearson’s *R* = −0.036, p = 0.78). On the other hand, the JSD derived from epitranscriptome profiles had an average value of 0.12 and demonstrated a significantly negative correlation with age (Pearson’s *R* = −0.29, p = 0.025). These findings suggested a greater inter-individual heterogeneity in the distribution of epitranscriptome profiles, compared to transcriptome, and this heterogeneity tended to decrease with age (Figure 5A). To further explore how NAD-RNAs were modulated with age and its consequent impact on the progression of aging, we analyzed NAD-RNA profiles from all age groups. Interestingly, even though NAD levels in circulating blood exhibited substantial inter-individual variations with advancing age,^22^ we found that the number of NAD capping events tended to increase in aged human subjects (Figure 5B). To dissect this observation, we grouped age-associated trajectories into two major clusters using hierarchical clustering (Figure 5C and Table S6). For genes in each cluster, the age-associated dynamic of their transcript abundances and NAD modification levels did not exhibit positive correlations, suggesting that the transcriptome and NAD-modified epitranscriptome entailed distinct facets of the aging process (Figures S3A and S3B). Furthermore, increased NAD modification was found for genes in cluster 1, with their function being involved in basic cellular events and adaptive immune response. NAD capping of genes associated with aminoacyl-tRNA biosynthesis and cellular respiration, which were ascribed as cluster 2, remained generally constant with age (Figure 5C and Table S7).

By inspecting the correlation between NAD modification and age, we identified a set of NAD-RNAs that highly associated with age (n = 96) (Figure 5D). Specifically, select NAD-RNAs, such as those involved in protein folding (PDIA3), and heat shock response (HSF2) had increased capping with age (Table S6), but the abundance at their RNA transcript levels was not increased (Figure 5E). In addition, NAD capping of genes linked to mRNA decay (UPF2), and calmodulin binding (NRGN) were decreased during aging (Figure 5E and Table S6). The expression levels of UPF2 and NRGN also decreased during aging (Figure 5E).

To validate age-associated changes of NAD-RNA, we utilized a quantitative PCR strategy slightly modified from the CapZyme-Seq method.^23^ We introduced three types of spike-in RNAs: 1) a synthetic NAD-capped RNA as the positive control, 2) a synthetic m^7^G-RNA as the negative control, and 3) a synthetic non-capped ppp-RNA as the baseline negative control. The RNA mixture was subjected to calf-intestinal alkaline phosphatase treatment to remove phosphate groups from the 5′ end of non-capped RNAs. Subsequently, NudC pyrophosphatase was introduced to hydrolyzed NAD-capped RNA, producing a ligatable 5′ monophosphate on these RNAs, followed by adapter ligation, adapter-specific cDNA synthesis, and PCR amplification (Figure S4A). Control experiment showed that NAD-capped spike-in RNA, but not ppp- and m^7^G-RNA, was selectively and significantly enriched (Figure S4B). We proceeded to validate the NAD capping of four endogenous transcripts, including two transcripts with age-increased NAD modification (PDIA3 and HSF2), and two with age-decreased NAD capping (UPF2 and NRGN). In support of our findings at the epitranscriptome level, we found that NAD capping of PDIA3 and HSF2 was dramatically higher in aged compared to young individuals. On the other hand, UPF2 and NRGN had a significant decline of NAD capping in aged individuals (Figure 5F).

### RNA-based prediction of chronological age

**Figure 6:**
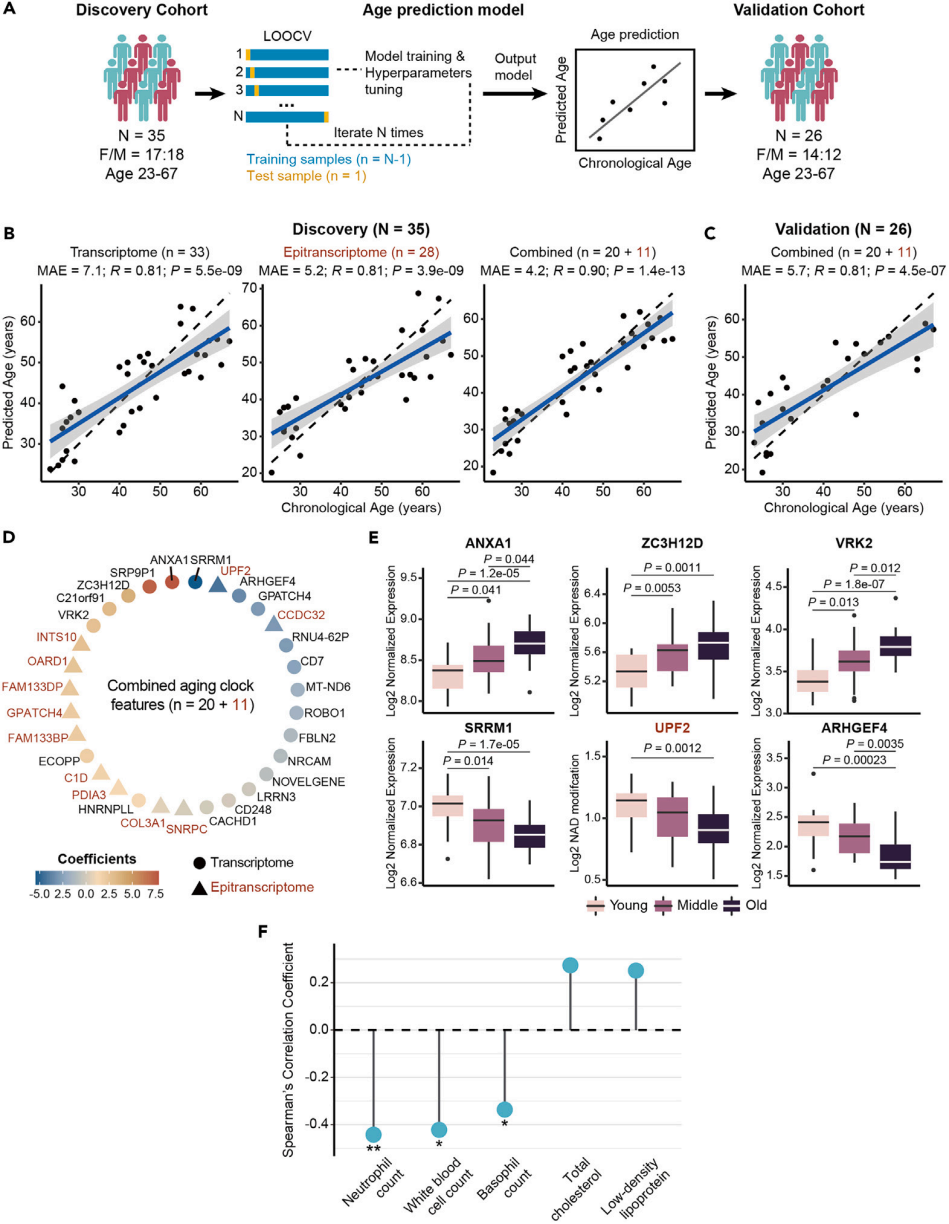
RNA-based age prediction model (A) Illustration of the development and validation of age prediction model. (B) Performance of age prediction models based on transcriptome (left), NAD-modified epitranscriptome (middle) and the combination of transcriptome and NAD-modified epitranscriptome (right) in the discovery cohort. (C) Performance of the combined age prediction model in the validation cohort. Each dot represents an individual. The blue solid line and shaded region represent a regression line and its 95% confidence intervals, respectively. The black dashed line represents the identity line. N is the number of individuals and n is the number of features. Numbers of epitranscriptomic features were colored in red. (D) Coefficients of features in the RNA-based aging clock that combined signatures from transcriptome and epitranscriptome. (E) Boxplot showing the age-associated trend of features contributing the most variance to the combined aging clock. Epitranscriptomic features were colored in red. (F) Age gap (predicted age – chronological age) was strongly associated with the blood cell count and blood cholesterol levels (|*R*_*s*_| > 0.25). p values were obtained with two-sided Spearman’s correlation test (∗p < 0.05, ∗∗p < 0.01).

Aging is a complex process that entails both functional and molecular changes. Currently, RNA-based aging clock solely relies on the longitudinal change of select RNA transcripts, which limits the accuracy of prediction.^24^ Given the fact that NAD-RNAs intrinsically link metabolite with gene expression, we reasoned that RNA-based clock, if combined with an additional layer of NAD-modified epitranscriptome, might substantially improve the performance of prediction. To do this, we randomly split the aging cohort into two groups: the discovery cohort (N = 35), which served as the training set, and the validation cohort (N = 26), which was used for model validation. Both cohorts were balanced for ages and gender distribution (Table S1). The performance of each model was subsequently evaluated by the median absolute error (MAE; unit, years) with leave-one-out cross-validation (Figure 6A). We found that the transcriptome-based model (MAE = 7.1; Pearson’s *R* = 0.81 and p = 5.5e-9) and epitranscriptome-based model (MAE = 5.2; Pearson’s *R* = 0.81 and p = 3.9e-9) could generate prediction of chronological age. We further combined signature from transcriptome and epitranscriptome, yielding an improved prediction model (MAE = 4.2; Pearson’s *R* = 0.90 and p = 1.4e-13) (Figure 6B and Tables S8, S9, and S10). To assess whether the trained model presented a dataset-specific bias, the combined model was applied to an independent validation cohort (N = 26). We obtained a comparable result from the validation cohort (MAE = 5.7; Pearson’s *R* = 0.81 and p = 4.5e-7), suggesting the model can be generalizable to the independent dataset (Figure 6C). By employing machine learning approaches, we confirmed that our aging clock, which combined both transcriptomic and epitranscriptomic signatures, exhibited better performance compared to the models solely based on the transcriptome or epitranscriptome (Figures S5A–S5F). These findings suggested that RNA-based aging clock with an additional epitranscriptomic layer could lead to improved age prediction accuracy. We assessed our model using elastic net regression (Figures 6B and 6C), which showed outperformed result than models based on ridge, lasso, and random forest regression (Figures S5A–S5F). Specifically, the features of combined aging clock were comprised of 20 transcriptomic and 11 epitranscriptomic signatures (Figure 6D). 16 of them positively correlated with the progression of age, such as transcriptomic signatures involved in innate immune response (ANXA1), cell cycle (ZC3H12D), and apoptosis (VRK2). On the other hand, 15 features negatively associated with the progression of age, such as transcriptomic signatures involved in RNA processing (SRRM1), GPCR pathway (ARHGEF4), and epitranscriptomic signatures involved in mRNA decay (UPF2) (Figure 6E). Moreover, we defined the age gap as subtracting real chronological age of human subjects from predicted age based on the combined aging clock, to investigate whether the accelerated (age gap >0) or decelerated biological ages (age gap <0) were associated with distinct physiological conditions. Using correlation analysis, we found that the advanced biological age was significantly associated with lower levels of blood cell count, such as neutrophil, white blood cell, and basophil, while positively correlated with blood cholesterol levels, including total cholesterol, and low-density lipoprotein (Figure 6F and Table S12). Together, these data highlight the potential of RNA-based aging clock for the prediction of chronological age and perhaps the physiological condition of individual subjects.

Combined, our study revealed the first NAD-modified epitranscriptome from human PBMCs. Using enONE, we were able to pinpoint epitranscriptomic alteration of NAD capping during the aging process. With a machine learning approach, we build an accurate RNA-based aging clock combing signatures from both transcriptome and epitranscriptome, which could predict the chronological age.

## Discussion

Incorporation of NAD, the hub metabolite and redox cofactor for cells, into RNA represents the crosstalk between metabolite and gene expression, defining a critical layer of epitranscriptomic regulation. NAD-RNA sequencing technologies, particularly our recently developed ONE-seq,^8^ have substantially facilitated the epitranscriptome-wide identification of novel NAD-capped RNAs across phyla.^16^ However, since these methods require affinity-based enrichment, data normalization simply borrowed from bulk RNA-seq could not be readily applied. In this study, we devise a general approach for epitranscriptome-wide NAD-RNA-seq data normalization and evaluation. Previous epitranscriptome profiling approaches have added low amount of ERCC spike-ins to account for the unwanted variation. However, ERCC spike-ins are only composed of 92 exogenous transcripts with length ranging from 250 to 2,000 nt, which cannot faithfully mimic the endogenous transcripts throughout stepwise enrichment procedures and library preparation.^14^^,^^25^ Since NAD capping is an evolutionary conserved mechanism,^16^ our strategy deliberately includes *Drosophila* total RNA that comprises full spectrum of NAD epitranscriptome as spike-in. Particularly, we introduce relatively higher amount of fly RNA than that of human as surrogate for RNA loss resulted from affinity-based enrichment procedures. Since *Drosophila* is evolutionarily apart from human,^26^ sequencing reads can be easily distinguished in the subsequent data analysis. Although scaling-based approaches have been extensively used in the analysis of epitranscriptome profiles, we demonstrate that integration of global scaling and RUV strategy can optimize normalization performance (see Figure S2). Selection of the “best” normalization, however, might not be feasible in practice due to the subjective definition of optimality.^27^^,^^28^ Therefore, enONE emphasizes the choice of an appropriate rather than the “best” strategy. Collectively, enONE can efficiently remove the impact of unwanted noises arising from affinity-based enrichment in the heterogeneous human aging data while preserving the age-associated signals. Also, we highlight the application that enONE, an open-source R package, can be extended into the analysis of other types of epitranscriptomic sequencing data that involve stepwise enrichment procedures, e.g., m^6^A-seq.

The enONE computational framework facilitates the identification of NAD-RNA biomarkers. As a source of liquid biopsy, peripheral blood can serve as sentinel tissue to monitor individual health in a non-invasive manner. Identification of novel blood-derived features may open up new avenues in detection of biomarkers for various physiological and pathological conditions. Here, we reveal prominent features of NAD-RNAs from human PBMCs. Large collections of NAD-RNAs are produced by protein-encoding genes, with their biological functions mainly involved in basic cellular events, such as translation, RNA metabolism, and transcription. Additionally, genes with specialized function, such as those involved in immune system, are found to be capped by NAD. Thus far, yet the function of NAD-RNAs remains elusive, their dynamic changes may provide insights into how individual subjects respond to perturbations, such as the aging process. In line with the decline of NAD metabolite,^29^^,^^30^ our recent mouse liver study has shown that the extent of NAD capping tends to decrease with age.^8^ However, in contrast to biological replicates from a controlled laboratory environment, this pattern appears to be much more complex in individual human subjects. A recent study based on a large Chinese cohort (n = 1,518) has shown that peripheral blood NAD levels demonstrate remarkable inter-individual heterogeneity and gender-specific variation during aging.^22^ In the present study, we propose that NAD capping in PBMCs tends to increase in elder population (see Figure 5B). This finding suggests that the addition or removal of 5′-terminus NAD moiety in different tissues might not be solely dependent on the cellular reserve of NAD. In addition, NAD-modified epitranscriptome might be remodeled by other mechanisms during aging, such as the activity of NAD-RNA decapping enzymes^31^ and small RNAs that destabilize NAD-capped transcripts.^32^ Moreover, our data illustrate some of the age-associated NAD-RNAs that are involved in molecular pathways profoundly impinging on the hallmarks of aging, such as mitochondrial function and immune system.^33^ During aging, mitochondrial function progressively deteriorates, compromising the homeostasis of energy metabolism and redox status.^33^ Previous studies have reported that mitochondrial genome can produce NAD-RNAs in various eukaryotic organisms, including yeast,^34^ *Arabidopsis*,^35^ and human.^36^ Here, our data reveal a substantial proportion of transcripts, including 30 out of the 37 mitochondria-encoded transcripts and 81 mitochondria-related genes from nucleus, can generate NAD-capped RNAs. Among them, we note an age-associated increase in NAD capping of transcripts associated with essential mitochondrial functions, such as oxidative phosphorylation. These findings suggest that NAD capping may provide novel insights into mechanisms that modulate mitochondrial activities with age. In addition, increased inflammation, characterized by an elevation of circulating inflammatory factors, is another key hallmark of aging.^33^ Our data highlight that the increase of NAD modification of genes related to adaptive immune system represents a new feature in the system-wide manifestation of inflammation during human aging.

Our preliminary study provides proof-of-concept evidence that blood-derived, RNA-based measures can be used to predict the chronological age of human. Additionally, our new RNA-based aging clock, which combines an additional layer of NAD-modified epitranscriptome, exhibits a better age prediction performance than those solely based on the abundance of transcripts, e.g., bulk RNA-seq^37^ and recently single-cell RNA-seq.^38^ Since there is currently no gold standard measure of biological aging,^39^ it is tempting to exploit how NAD-capped RNAs as biological implications might signify physiological and perhaps pathological conditions.

Taken together, we propose enONE as a flexible, modular, and general framework for the spike-in-based normalization and evaluation of NAD-RNA sequencing data in a wide spectrum of biological contexts. To the best of our knowledge, enONE is the first computational approach for NAD-modified epitranscriptome profiles, which can be extended into the analysis of other types of epitranscriptomic sequencing data that involve stepwise enrichment procedures, e.g., m^6^A-seq. Using enONE, we identify hundreds of NAD-capped transcripts from human peripheral blood and reveal age-associated dynamics of NAD capping that are linked to biological processes impinging on aging hallmarks. Potentially, blood-derived NAD-RNAs can be exploited as non-invasive biomarkers for aging.

### Limitations of the study

The present study mainly focusses on disentangling the age-associated features of NAD-capped epitranscriptome from human peripheral blood using enONE. However, the overall number of NAD-RNAs from individual subjects is not simply correlated with age (see Figure 5B), raising the possibility that additional age-associated features remain to be interrogated. For instance, gender effect has been shown to impact the fundamental metabolism^40^ and gene expression programs.^41^^,^^42^ How NAD-capped RNAs, which represent the crosstalk between the metabolome and transcriptome, vary between different genders warrants future investigation. In addition, our human aging cohort is a relatively small-scale population recruited from the local community of Shanghai, which does not represent the general population. That how this set of RNA biomarkers compares with existing DNA-based quantification is worth of future investigation in a larger and more diverse cohort.^43^

## STAR★Methods

### Key resources table

**Table:** 

REAGENT or RESOURCE	SOURCE	IDENTIFIER
**Biological samples**		

*D. melanogaster*: 5905	Bloomington Drosophila Stock Center	RRID: BDSC_5905

**Chemicals, peptides, and recombinant proteins**		

yDcps	New England Biolabs	M0463S
Glycoblue	Thermo Fisher Scientific	AM9516
RRI	Takara Bio	2313A
TRizol	Takara Bio	9109
CIAP	Thermo Fisher Scientific	18009–027
NudC Pyrophosphatase	New England Biolabs	M0607S
T4 RNA Ligase 1 (ssRNA Ligase)	New England Biolabs	M0204S

**Critical commercial assays**		

Fast RNA-seq Lib Prep Kit V2	Abclonal	RK20306
ABScript II cDNA First-Strand Synthesis Kit	Abclonal	RK20400
ABScript III RT Master Mix for qPCR	Abclonal	RK20428
MEGAscript T7 Transcription Kit	Thermo Fisher Scientific	AM1334

**Deposited data**		

Raw and analyzed data	Gene Expression Omnibus	GSE226636

**Experimental models: Organisms/strains**		

*D. melanogaster*: 5905	Bloomington Drosophila Stock Center	RRID: BDSC_5905

**Oligonucleotides**		

List of primers and other oligonucleotides, see Table S13	This paper	N/A

**Software and algorithms**		

enONE	This paper	https://github.com/thereallda/enONE

### Resource availability

#### Lead contact

Additional information and requests for resources and reagents used in this study should be directed to and will be fulfilled by the lead contact, Nan Liu, (liunan@sioc.ac.cn).

#### Materials availability

This study did not generate new unique reagents.

#### Data and code availability

• All high-throughput RNA sequencing data as well as transcript quantifications have been deposited at the Gene Expression Omnibus under accession number GSE226636.
• All original code has been deposited at Zenodo (https://doi.org/10.5281/zenodo.10068529). enONE is available as an R package on GitHub (https://github.com/thereallda/enONE). The datasets and the code are publicly available as of the date of publications.
• Any additional information required to reanalyze the data reported in this paper is available from the lead contact upon request.

### Experimental model and study participant details

The project was approved by the Medical Ethics Committee of Shanghai Changzheng Hospital (2022SL006). Informed consent was obtained from all subjects in accordance with the local research Ethics Committee guidelines. We conducted a cross-sectional study of natural aging in community subjects recruited from Shanghai Chang Zheng Hospital. The aging cohort consists of 31 females and 30 males, aged from 23 to 67 years old. Details of participant information were listed in Table S1. Inclusion criteria for cohort were: 1) above 20 years old; 2) independently able to provide written informed consent. For comparative analysis of age-related subgroups, the cohort was divided into three age groups: Young (20–35 years old), Middle (36–50 years old), and Old (51 years old and above). Height and weight are measured by trained staff following standardized protocols. BMI (kg/m^2^) is derived from the calculation and stored for further analyses. After 5 minutes of rest in the seated position, blood pressure (mmHg) was measured three times with an automatic sphygmomanometer and the mean of the measurements was used for analysis. Blood samples were taken from all patients in the morning after they had been seated for 5 minutes. All participants had blood drawn using lithium heparin tubes (BD Vacutainer, catalog: 367884) by phlebotomists and consented to having their de-identified survey data made publicly available.

### Method details

#### Isolation of peripheral blood mononuclear cells (PBMCs) from whole blood

For isolation of peripheral blood mononuclear cells (PBMCs), 5 mL whole blood was mixed with 630 μL OptiPrep (Sigma-Aldrich, catalog: D1556) and 500 μL solution C (0.85% (w/x) NaCl and 10 mM Tris-HCl, pH 7.4), followed by centrifugation at 4°C and 1,300 g for 30 min. PBMCs were collected and mixed with two volumes of solution C, followed by centrifugation at 4°C and 500 g for 10 min. Cell pellet was washed twice with 1 mL PBS. The suspension was used for NAD-RNA detection.

#### *In vitro* transcription of NAD-RNA, and m^7^G-RNA

To assess the sensitivity of enrichment procedures, spike-in NAD-RNA (500 nt; sequence A) and m^7^G-RNA (500 nt; sequence A) with identical sequence, oligonucleotide without adenine was synthesized (Genewiz) and were subjected to polyadenylation for poly(A) tails elongation (Table S13). To assess the specificity of enrichment procedures, spike-in m^7^G-RNA (500 nt; sequence B) oligonucleotide was synthesized (Genewiz) and were subjected to polyadenylation for poly(A) tails elongation (Table S13). To prepare the baseline control for qPCR, spike-in ppp-RNA (500 nt) oligonucleotide was synthesized (Genewiz) and were subjected to polyadenylation for poly(A) tails elongation (Table S13). To prepare the positive control for qPCR, spike-in NAD-RNA (500 nt) oligonucleotide was synthesized (Genewiz) and were subjected to polyadenylation for poly(A) tails elongation (Table S13). For *in vitro* transcription, 10 μM of double-stranded DNA (dsDNA) template in 100 μL transcription buffer (Promega, catalog: P1300), along with 1 mM of each of GTP, CTP and UTP, with 4 mM NAD (for NAD-RNA) or 4 mM m^7^GpppA (New England Biolabs, catalog: S1406S) (for m^7^G-RNA), 10 μL of T7 RNA polymerase (Promega, catalog: P1300), 5% DMSO, 5 mM DTT and 2.5-unit RNase inhibitor were added and the transcription mixture was incubated at 37°C for 4 h. The reaction was incubated with 11-unit DNase I (Promega, catalog: P1300) at 37°C for 30 min to remove the DNA template. RNA was then extracted using acid phenol/chloroform and precipitated with isopropanol (with 0.3 M sodium acetate, pH 5.5) at −80°C overnight. The RNA pellet was washed twice with 75% ethanol, air-dried, re-dissolved in DEPC-treated H_2_O, and stored at −80°C.

#### NAD-capped RNA sequencing

Total RNAs from human PBMCs and total RNAs from *Drosophila* (spike-in) were prepared in accordance with the manufacturer’s instruction (Takara Bio, catalog: 9108). Total RNAs (10 μg) from human PBMCs were mixed with 40 μg *Drosophila* RNA (spike-in 1), 0.1 ng synthetic RNAs (spike-in 2: 5% NAD-RNA/95% m^7^G-RNA; sequence A), and 0.1 ng synthetic RNAs (spike-in 3: 100% m^7^G-RNA; sequence B). The mixture of total RNAs and spike-in RNAs was incubated with 100 mM HEEB (1 M stock in DMSO) with ADPRC (25 μg/mL) in 100 μL of ADPRC reaction buffer (50 mM Na-HEPES pH 7.0, 5 mM MgCl_2_) at 37°C for 1 h. 100 μL of DEPC-treated H_2_O was then added and acid phenol/ether extraction was performed to stop the reaction. RNAs were precipitated by ethanol and re-dissolved in 100 μL of DEPC-treated H_2_O. 5 μL of biotinylated RNAs were kept as input. After HEEB reaction, biotinylated RNAs were incubated with streptavidin bead particles (6 μL, MedChemExpress, catalog: HY-K0208) and 0.4 U/μL of RNase Inhibitor (Takara Bio, catalog: 2313B) at 25°C for 30 min. Beads were washed four times with streptavidin wash buffer (50 mM Tris-HCl (pH 7.4) and 8 M urea), and three times with DEPC-treated H_2_O. To ensure complete elution, biotin-conjugated RNAs were replaced from streptavidin beads by incubating with 1 mM biotin buffer (20 μL NaOH pH 8.0, Sigma-Aldrich, catalog: B4639) at 94°C for 8 min, followed by incubation with 500 nM NudC (New England Biolabs, catalog: M0607S) in 25 μL of NudC reaction buffer (100 mM NaCl, 50 mM Tris-HCl pH 7.9, 10 mM MgCl_2_, 100 μg/ml Recombinant Albumin) at 37°C for 30 min. After NudC treatment, biotinylated-RNAs that are resistant to NudC catalysis, potentially derived from contaminating m^7^G-RNAs, were retained on beads by incubation with high-capacity streptavidin particle (20 μL, Thermo Fisher Scientific, catalog: 20357) at 25°C for 30 min. Eluted RNAs in the supernatant were used for next step. Input (see above) and NudC-eluted RNAs were used for NGS library construction, in accordance with the manufacturer’s instructions (mRNA-seq Lib Prep Kit for Illumina, Abclonal, catalog: RK20302). Library quality was assessed by Bioanalyzer 2100 (Agilent, United States), and quantification was performed by qRT-PCR with a reference to a standard library. Libraries were pooled together in equimolar amounts to a final 2 nM concentration and denatured with 0.1 M NaOH (Sigma, catalog: 72068). Libraries were sequenced on the Illumina NovaSeq 6000 system (paired end; 150 bp).

#### Validation of NAD-capped RNA by a modified CapZyme-Seq method

Total RNAs (3 μg) from human PBMCs of young (n = 3) and old (n = 3) individuals were mixed with *Drosophila* total RNA (7 μg), as surrogate for RNA loss, and polyadenylated spike-in RNAs generated from different sequence templates, including 0.1 ng NAD-RNA (500 nt), 0.1 ng m^7^Gppp-RNA (500 nt), and 0.1 ng ppp-RNA (500 nt). RNAs were incubated with 10 U CIAP (Thermo Fisher Scientific, catalog: 18009019) in the presence of 40 U RNaseOUT at 37°C for 1 h to remove 5′-terminal phosphate from non-capped RNAs. After purification with RNAiso Plus (Takara Bio, catalog: 9108), CIAP-treated RNAs were incubated with 250 nM NudC (New England Biolabs, catalog: M0607S) in 40 μl of NudC reaction buffer (100 mM NaCl, 50 mM Tris–HCl pH 7.9, 10 mM MgCl2, 100 μg/ml Recombinant Albumin, 40 U RNaseOUT) at 37°C for 30 min. This step aims to hydrolyze the cap from NAD-RNA resulting in ligatible monophosphate at the 5′ end. Products were purified with RNAiso Plus (Takara Bio, catalog: 9108) and further ligated with 100 μM 5′ adaptor oligo listed in Table S13, in the presence of 15 U T4 RNA ligase 1 (New England Biolabs, catalog: M0202), 15% of PEG8000, 1 mM ATP and 40 U RNaseOUT in 40 μl of 1× T4 RNA ligase buffer at 16°C for 16 h. RNAs were purified and re-dissolved in 20 μl of DEPC-treated H_2_O. Reverse transcription was performed with designed primer listed in Table S13, by ABScript II cDNA First-Strand Synthesis Kit (ABclonal, catalog: RK20400). 10 μl of products was diluted to 200 μl and served as input. Meanwhile, 5 μl of reversely transcribed products were further amplified by PCR using adaptor-specific primers listed in Table S13. The PCR program operated with an initial denaturation step of 3 min at 95°C, amplification for 14 cycles (denaturation for 15 s at 95°C, annealing for 15 s at 60°C and extension for 6 min at 72°C), and a final extension for 5 min at 72°C. The ppp-RNA (500 nt) was used as the baseline control, with NAD-RNA (500 nt) serving as the positive control, and m^7^G-RNA (500 nt) serving as the negative control. Primers were listed in Table S13. To calculate the enrichment from qRT-PCR data, the Ct value of the target gene was first normalized to the Ct of the ppp-RNA (baseline). Next, the normalized PCR+ fraction value (ΔCt of the target gene normalized to the ppp-RNA) was normalized to the background (ΔCt calculation for the gene in the input), to yield the ΔΔCt value. The linear conversion of this ΔΔCt resulted in the fold enrichment. Significance was assessed by Student’s *t* test.

### Quantification and statistical analysis

#### High-throughput sequencing data analysis

All sequencing reads were processed with Trim Galore (v0.6.6)^44^ with the parameters “--nextseq 30 --paired” to remove the adapter sequences (AGATCGGAAGAGC) from NovaSeq-platforms, and reads longer than 20 bp were kept. Reads that passed the quality control procedure were kept and mapped to the combined genome of *Homo sapiens* (GRCh38) and *Drosophila melanogaster* (dmel-all-chromosome-r6.36) using STAR (v2.7.6a)^45^ with default parameters. Uniquely mapped read pairs were counted against annotations from *Homo sapiens* (Ensembl: GRCh38.94) and *Drosophila melanogaster* (Flybase: dmel-all-r6.36) using featureCounts (v2.0.1)^46^ with parameters “-p -B -C” and summarized as gene-level counts. Sequencing saturation was assessed by randomly subsampling the original libraries and examined the corresponding changes in the number of genes, derived from human genome, with more than 10 read counts.

#### enONE workflow

enONE is implemented in R and publicly available at https://github.com/thereallda/enONE. enONE workflow consists of four steps: 1. Quality control; 2. Gene set selection; 3. Normalization procedures; 4. Normalization performance assessment. By “log” transformation, we generally refer to the log_2_(x+1) function unless otherwise stated. Below, steps are shown in detail.

##### Quality control

The goal of quality control was to remove problematic or noisy observations from downstream analysis. In this study, we used sample and gene filtering to control data quality. To assess outliers, we applied Rosner’s outlier test on principal component 1. Principle component analysis (PCA) was performed with prcomp function on the top 20,000 genes based on a transformed counts matrix by vst function from R package DESeq2 (v1.36.0).^13^ All samples were kept for subsequent analysis. To keep well-detected genes across samples, we used filterByExpr function from the R package edgeR (v3.38.4)^47^ with parameter “min.count=20”. All ribosomal RNA encoded genes and TEC genes were excluded.

##### Gene set selection

enONE defined three sets of control genes for adjustment of the unwanted variations, evaluation of the unwanted variations, and evaluation of the wanted variations, respectively. For adjustment of the unwanted variation, we defined the 1,000 least significantly enriched genes (denoted as anchor set) in *Drosophila* spike-ins, ranked by likelihood ratio, as negative control genes. Since the effect of affinity-based enrichment was the known covariate of interest, we used these genes, that were not affected by the enrichment effect, to compute the unwanted variation in the subsequent RUV procedure. For evaluation of the unwanted variation, we defined the 500 least significantly varied genes in human, ranked by likelihood ratio, as negative evaluation genes. We performed differential analysis test across all covariates of interest to determine genes with constant expression levels. Since the variation of constant genes could reflect the handling effects, those genes could be used to evaluate the removal of unwanted variation in the subsequent normalization evaluation step. For evaluation of the wanted variation, we defined the 500 most significantly enriched genes in human, ranked by likelihood ratio, as positive evaluation genes. We performed differential analysis test between enrichment and input samples in human to determine genes affected by enrichment procedures. Those genes were used to evaluate the preservation of wanted variation in the subsequent normalization evaluation step.

##### Normalization procedures

enONE implemented global scaling and regression-based methods for the generation of two-part normalization schemes. For the global scaling normalization methods, gene-level read counts were adjusted by a sample-wise scale factor. This procedure encompassed five distinct scaling methods, including: 1) Total Count (TC): The scale factor was defined as the sum of the read counts across all genes within a sample. 2) Upper-Quartile (UQ): The scale factor was computed as the upper-quartile of the gene-level count distribution for each sample. 3) TMM^12^: The scale factor was determined using a robust estimation of the overall expression fold change between a given sample and a reference sample. TMM was performed by the calcNormFactors function from R package edgeR with parameter: method = “TMM”. The default criterion for reference sample selection was based on its upper quartile being the closest to the mean upper quartile value across all samples. 4) DESeq^13^: The scale factor for a given sample was calculated as the median fold-change between the samples and a pseudo-reference sample, whose counts were defined as the geometric means of the counts across samples. This method was performed by the calcNormFactors function from R package edgeR with parameter: method = “RLE”. Note that the method discarded any gene having zero count in at least one sample. 5) PoissonSeq: The scale factor was derived based on an iterative estimate of sequencing depth, considering a subset of genes characterized by Poisson goodness-of-fit statistics.^17^ By default, this method discarded genes with less than 5 read counts.

For the regression-based procedures, enONE considered the following generalized linear model, which allows adjustment for factors of unwanted variation:

(Equation 1) Y=Xβ+Wα+O,

where *Y* was the *n* x *j* matrix of gene-level read counts, *X* was an *n* x *m* design matrix corresponding to the *m* covariates of interest of ‘‘wanted variation’’ (e.g., treatment) and *β* its associated *m* x *j* matrix of parameters of interest, *W* was an *n* x *k* matrix corresponding to unknown factors of unwanted variation and *α* its associated *k* x *j* matrix of nuisance parameters, *O* was an *n* x *j* matrix of offsets that can either be set to zero or estimated with other normalization procedure (such as TMM normalization). Here, *n* is the number of genes, *j* is the number of samples, *m* is the number of covariates, and *k* is the chosen number of unwanted variation factors.

The unwanted variation *W* was estimated via singular value decomposition (SVD) using approaches implemented in R package RUVSeq (v1.30.0).^14^ In this study, we introduced three variants of RUV, including: 1) RUVg: This variant estimated the unwanted variation factors based on negative control genes that were assumed to exhibit constant expression across all samples (*β* = 0). 2) RUVs: This method estimated the factors of unwanted variation based on negative control genes derived from the replicate samples, such as those within each treatment group, where the covariates of interest remained constant (*β* = 0). 3) RUVse: This novel variant was a modified version of RUVs. It estimated the unwanted variation factors using negative control genes from the replicate samples within each assay group (i.e., enrichment and input), with the assumption that the enrichment effect remained constant. Following the estimation of unwanted variations, for a chosen value of *k* (1 ≤ *k* ≤ *j*), the adjusted read counts *Y∗* can be defined by $Y^{*}=Y-{\hat{W}}^{\left(k\right)}{\hat{\alpha }}^{\left(k\right)}-O\text{.}$

##### Normalization performance evaluation

To evaluate the performance of normalization, we leveraged eight normalization performance metrics that related to different aspects of gene expression measures.^27^

The following four metrics evaluated normalization procedures based on how well the samples can be clustered according to factors of wanted or unwanted variation. Clustering by wanted factors was desirable, while clustering by unwanted factors was undesirable. We used silhouette widths as clustering measurements. For any clustering of *n* samples, the silhouette width of sample *i* was defined as:

(Equation 2) $$sil\left(i\right)=\frac{b\left(i\right)-a\left(i\right)}{\max\left\{a\left(i\right),b\left(i\right)\right\}}\in \left[-1,1\right]\text{,}$$

where *a(i)* denoted the average distance (by default, Euclidean distance over the first three PCs of expression measures) between the *i*th sample and all other samples in the cluster to which *i* was assigned and *b(i)* denoted the minimum average distance between the *i*th sample and samples in other clusters. The larger the silhouette widths, the better the clustering. Thus, the average silhouette width across all *n* samples provides an overall quality measure for a given clustering. Silhouette width was calculated with the silhouette function from R package cluster (v2.1.3). These metrics were defined as follows: 1) BIO_SIM: Group the *n* samples according to the value of a categorical covariate of interest (e.g., age group or treatment) and compute the average silhouette width for the resulting clustering. 2) BATCH_SIM: Group the *n* samples according to the batch and compute the average silhouette width for the resulting clustering. 3) EN_SIM: Group the *n* samples according to the assay (e.g., enrichment or input) and compute the average silhouette width for the resulting clustering. 4) PAM_SIM: Cluster the *n* samples using partitioning around medoids (PAM) for a range of numbers of clusters and compute the maximum average silhouette width for these clusters. PAM clustering was done by the pamk function from R package fpc (v2.2-9). Large values of BIO_SIM, EN_SIM and PAM_SIM and low values of BATCH_SIM were desirable.

The next two metrics evaluated the association of log-count principal components (by default, the first 3 PCs) with “evaluation” principal components of wanted or unwanted variation. 1) UV_COR: The weighted coefficient of determination ${\bar{R}}^{2}$ for the regression of log-count principal components on factors of unwanted variation (UV) derived from negative evaluation genes. The submatrix of log-transformed unnormalized counts for negative evaluation genes is row-centered and scaled and factors of unwanted variation are defined as the right-singular vectors as computed by the svd function. 2) WV_COR: The weighted coefficient of determination ${\bar{R}}^{2}$ for the regression of log-count principal components on factors of wanted variation (WV) derived from positive evaluation genes. The WV factors were computed in the same way as the UV factors above, but with positive instead of negative evaluation genes. Large values of WV_COR and low values of UV_COR were desirable.

The weighted coefficients of determination were computed as follows. For each type of evaluation criterion (i.e., UV, or WV), regressed each expression PC on all supplied evaluation PCs. Denoted *SST*_*k*_ as the total sum of squares, *SSR*_*k*_ as the regression sum of squares, and *SSE*_*k*_ as the residual sum of squares for the regression for the *k*th expression PC. The coefficient of determination was defined as:

(Equation 3) $$R_{k}^{2}=\frac{{SSR}_{k}}{{SST}_{k}}=1-\frac{{SSE}_{k}}{{SST}_{k}}\text{,}$$

and weighted average coefficient of determination as:

(Equation 4) $${\bar{R}}^{2}=\frac{\sum_{k}{SST}_{k}R_{k}^{2}}{\sum_{k}{SST}_{k}}=\frac{\sum_{k}{SSR}_{k}}{\sum_{k}{SST}_{k}}=1-\frac{\sum_{k}{SSE}_{k}}{\sum_{k}{SST}_{k}}\text{,}$$

The next two metrics evaluated the similarity of gene expression distributions. We defined gene-level relative log-expression (RLE) measures, as log-ratios of read counts to median read counts across samples, for comparing distribution of gene expression. RLE was defined as follows:

(Equation 5) $${RLE}_{ij}=\log\frac{Y_{ij}}{{Median}_{i}Y_{ij}}\text{,}$$

for gene *i* in sample *j*. For similar distributions, the RLE should be centered around zero and have a similar distribution across samples. Thus, the metrics were defined as follows: 1) RLE_MED: Mean squared median RLE. 2) RLE_IQR: Variance of inter-quartile range (IQR) of RLE. Low values of RLE_MED and RLE_IQR were desirable.

In the enONE framework, the expression measures were normalized according to a set of methods (including raw counts) and the eight metrics above were computed for each dataset. The performance assessment results can be visualized using biplots and the normalization procedures were ranked based on a function of the performance metrics. In particular, enONE defined a performance score by orienting the metrics (multiplying by ±1) so that large values correspond to good performance, ranking procedures by each metric, and averaging the ranks across metrics.

#### Comparison methods

For benchmarking, we compared the enONE procedure with the regression-based method, RUVg, and the scaling-based approach, RADAR. For RUVg, we used the anchor set from *Drosophila* spike-ins as negative control genes and the first four estimated factors of unwanted variations for adjustment. For RADAR, we normalized the input libraries using the median-of-ratio method of DESeq based on input gene-level counts. To normalize the enrichment libraries, we estimated sample-wise size factors from the fold change $F_{n,j}=\frac{{en}_{n,j}}{{in}_{n,j}}$ of the top 1% genes ranked by enrichment read counts, where ${en}_{n,j}$ is enrichment read count and ${in}_{n,j}$ is the DESeq normalized gene counts from gene *n* and sample *j*.

#### Linear regression for normalization performance evaluation

*R*^*2*^ values of fitted linear models were used to quantify the strength of the linear relationships between a source of unwanted variation, i.e., batch effect, and global sample summary statistics, such as the first k PCs (1 ≤ *k* ≤ 6).^48^ The lm R function was used for this analysis.

#### Spearman correlation

Spearman correlation coefficients were used to determine the monotonic relationships between a source of unwanted variation, i.e., batch effect, and the raw or normalized gene-level read counts. The cor R function was used for this analysis.

#### ANOVA

To evaluate the effects of the different sources of effect on the data, ANOVA were performed. For evaluating the batch effect, ANOVA tests were computed across batches. Genes with p value <0.01 were deemed to be affected by the batch effect. The aov R function was used for this analysis.

#### Vector correlation

The Rozeboom squared vector correlation^49^ was used to quantify the strength of relationships between the first *k* expression PCs (1 ≤ *k* ≤ 6) and the enrichment covariates.

#### Adjusted rand index

The adjusted Rand index (ARI), a correction of the Rand Index, measures the similarity between two clustering.^50^ The ARI was used to evaluate the performance of normalization methods in terms of separation between enrichment and input samples. The first three expression PCs was utilized to perform ARI analysis.

#### Jensen-Shannon distance

To quantify differences in gene expression and NAD-RNA profiles between individuals, we calculated the pairwise distance for all individuals and their corresponding centroid using the Jensen-Shannon Distance.^20^^,^^21^ We first computed the centroid distribution *P*_*c*_ by averaging the transcriptomic or epitranscriptomic profiles from all samples, respectively. For each individual’s transcriptomic or epitranscriptomic distribution, the Jensen-Shannon Divergence (JSD) can be calculated as:

(Equation 6) $$JSD\left(P_{c},P_{i}\right)=H\left(\frac{P_{c}+P_{i}}{2}\right)-\frac{H\left(P_{c}\right)+H\left(P_{i}\right)}{2}\text{,}$$

where *P*_*i*_ is the distribution for individual *i* and *H* is the Shannon entropy function:

(Equation 7) $$H\left(X\right)=-\sum_{i=1}^{n}P\left(x_{i}\right)\log_{2}\left(P\left(x_{i}\right)\right)\text{,}$$

Finally, the Jensen-Shannon Distance can be calculated as the square root of JSD.

#### Quantitative NAD-RNA-seq data analysis

To identify NAD-RNA from sequencing data, we performed differential analysis using FindEnrichment function from enONE package. Significance of logarithmic fold changes was determined by a likelihood ratio test to approximate p values, and genes were adjusted for multiple testing using the Benjamini-Hochberg procedure to yield a false discovery rate (FDR). NAD-RNAs were defined as fold change of normalized transcript counts ≥2 and FDR <0.05 in enrichment samples compared to those in input samples. The fold change of normalized counts between pairwise enrichment and input sample was denoted as NAD modification level. Gene annotation information, such as chromosome, gene types, and gene lengths were retrieved from Ensembl (GRCh38.94) annotations. The violin plot, boxplot, bar plot, line chart and scatterplot were generated by R package ggplot2 (v3.3.6).^51^

#### RNA secondary structure analysis

For each transcript with 5′ untranslated region (UTR), the sequence of 100 bp region downstream of transcription start site (TSS) was used to calculate RNA minimum free energy (MFE) by ViennaRNA with default parameter.^19^ To generate a background distribution of MFE, transcripts that did not produce NAD-cap were randomly sampled 1,000 times from the whole transcriptome with a similar size (n = 600) to transcripts producing NAD-cap (n = 589).

#### Pathway enrichment analysis

Pathway enrichment analysis was performed using Metascape (v3.5).^52^ Pathways were defined as the molecular pathways of Reactome, the biological process (BP) of GO, the Kyoto Encyclopedia of Genes and Genomes (KEGG), the WikiPathways (WP), the canonical pathways of MSigDb, and the CORUM database. Metascape clustered enriched terms into non-redundant groups based on similarities between terms and used the most significant term within each cluster to represent the cluster. The resulting clusters of pathways were manually reviewed. Enrichment was tested using the hypergeometric test. Multiple hypothesis correction was performed with Benjamini-Hochberg procedure, and the significance threshold was defined as *α* = 0.05.

#### Hierarchical clustering analysis

Hierarchical clustering with a Euclidean distance metric was performed for both rows (NAD-RNAs, complete method) and columns (samples, ward’s method) on the scaled NAD modification matrix. Clustering and corresponding heatmaps were generated by R package Complex Heatmap (v2.12.1).^53^ Using cutree function with “k = 2” parameter, we identified 3 clusters of NAD-RNAs changing with age, ranging from 201 to 608 NAD-RNAs.

#### Trajectory analysis

To estimate NAD modification trajectories during aging, log NAD modification levels were *Z*-scored, and LOESS regression was fitted for each gene. Similarly, log expression levels were used to estimate the gene expression trajectories during aging. The trajectory of clusters was estimated using the median levels of genes in each cluster. Pathways were queried as above to gain insights into the biological functions of each cluster. Top five non-redundant enriched terms were shown. To identify NAD-RNAs that correlated with age, we computed Spearman’s rank correlation coefficients (*R*_*s*_) for each NAD-RNAs on the basis of NAD modification and age. The resulting correlation metrics was then filtered, such that only NAD-RNAs that were significantly correlated with age (p < 0.05 and | *R*_*s*_ | ≥ 0.3) were considered. Similarly, correlation between age and gene expression levels was computed.

#### Age prediction model

We developed different age prediction models for human PBMCs based on NAD-modified epitranscriptome, transcriptome, and the combination of NAD-modified epitranscriptome and transcriptome, respectively. To construct those models, we employed four machine learning approaches, including elastic net, lasso, ridge, and random forest regression. Aging cohort was randomly split into the discovery cohort (N = 35) for model training, and the validation cohort (N = 26) for independent evaluation. Age prediction was performed by regressing chronological age on log-transformed feature levels (i.e., NAD modification and transcript abundances) using the discovery cohort. To build a robust aging clock, we pre-selected features using the age-correlated NAD-RNAs (p < 0.05 and | *R*_*s*_ | ≥ 0.3) and transcripts (p < 0.05 and | *R*_*s*_ | ≥ 0.5) as a starting point. To begin, we randomly split the discovery cohort into training (70%) and test (30%) sets with balanced ages. Model optimization including hyperparameter tuning was done by a grid search with leave-one-out cross-validation (LOOCV) based on training sets. Model performance was assessed on test sets, using several statistics including median absolute error (MAE), Pearson’s correlation coefficient and its associated p-value. Furthermore, we performed a cross-validation scheme for arriving the least biased estimates of the accuracy of the aging clocks, consisting of leaving out a single sample from the regression, predicting age for that sample, and iterating over all samples on discovery cohort. The best-tuned hyperparameters of trained models were listed in Table S11. Above model training and hyperparameter tuning are performed with R packages caret (v6.0-93), glmnet (v4.1-4) and randomForest (v4.7–1.1).

#### Association of age gap index and physiological phenotypes

Subtracting chronological age from predicted age, termed as the age gap, provided a normalized measure of the extent to which an individual appeared older (age gap >0) or younger (age gap <0) than same-aged peers.^54^ Then, we explored whether age gap was associated with physiological phenotypes, such as blood pressure, cholesterol levels, blood cell counts. Associations were evaluated using spearman’s correlation coefficients, while adjusting for chronological age and gender through linear regression. The associations of age gap and physiological phenotypes were listed in Table S12.

#### Statistical analyses

All p values reported herein were calculated using the non-parametric Mann-Whitney rank test unless otherwise stated.

## Supplemental information

Document S1. Figures S1–S5

Table S1. Cohort Demographics, related to Figure 2

Table S2. Lists of newly-identified NAD-RNAs in PBMCs of young adults (n = 556), related to Figure 4

Table S3. Lists of newly-identified NAD-RNAs in PBMCs of middle adults (n = 683), related to Figure 4

Table S4. Lists of newly-identified NAD-RNAs in PBMCs of old adults (n = 695), related to Figure 4

Table S5. Top 20 summarized pathways of NAD-capped RNAs in human PBMCs across all age groups (n = 809), related to Figure 4

Table S6. Lists of NAD-RNAs in each age-associated cluster, related to Figure 5

Table S7. Selected summarized pathways of each age-associated cluster based on NAD-modified epitranscriptome, related to Figure 5

Table S8. Age prediction model based on transcriptome using elastic net regression, related to Figure 6

Table S9. Age prediction model based on NAD-modified epitranscriptome using elastic net regression, related to Figure 6

Table S10. Age prediction model based on transcriptome and NAD-modified epitranscriptome using elastic net regression, related to Figure 6

Table S11. The best-tuned hyperparameters of age prediction models, related to Figures 6 and S5

Table S12. Associations between age gap and physiological phenotypes, related to Figure 6

Table S13. Sequences of qRT-PCR primers and other nucleotides used in this study, related to Figures 5, S1, and S4
