# Supplementary material for: Epitranscriptome analysis of NAD-capped RNA by spike-in-based normalization and prediction of chronological age
Source: iScience. 2023 Nov 22;26(12):108558. doi: 10.1016/j.isci.2023.108558 (PMC10716591; doi:10.1016/j.isci.2023.108558)
Supplement: Document S1. Figures S1–S5 [file mmc1.pdf]

iScience, Volume *26*

Supplemental information

Epitranscriptome analysis of NAD-capped RNA by spike-in-based normalization and prediction of chronological age

Dean Li, Shuwen Ge, Yandong Liu, Miaomiao Pan, Xueting Wang, Guojing Han, Sili Zou, Rui Liu, Kongyan Niu, Chao Zhao, Nan Liu, and Lefeng Qu

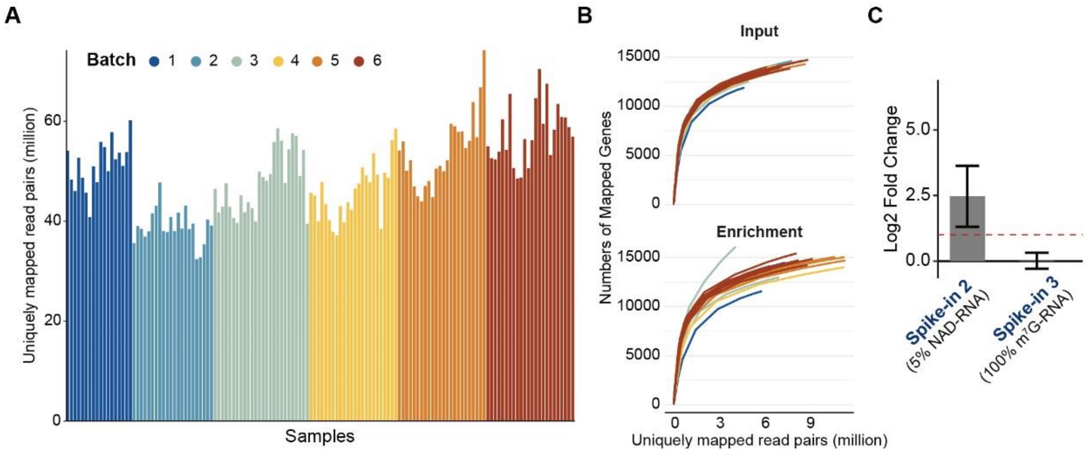

Figure S1 related to Figure 2, 3, and 4: Quality assessment of NAD-RNA-seq data. (A) Analysis of sequences alignment from human PBMCs. (B) Analysis of sequencing saturation for the human genome. (C) Barplot showing the fold change between enrichment and input samples of synthetic spike-ins with 5% NAD-caps and that with 100% m^7G-caps. Red dashed line represents the 2-fold enrichment cutoff. Data are mean ± s.e.m.

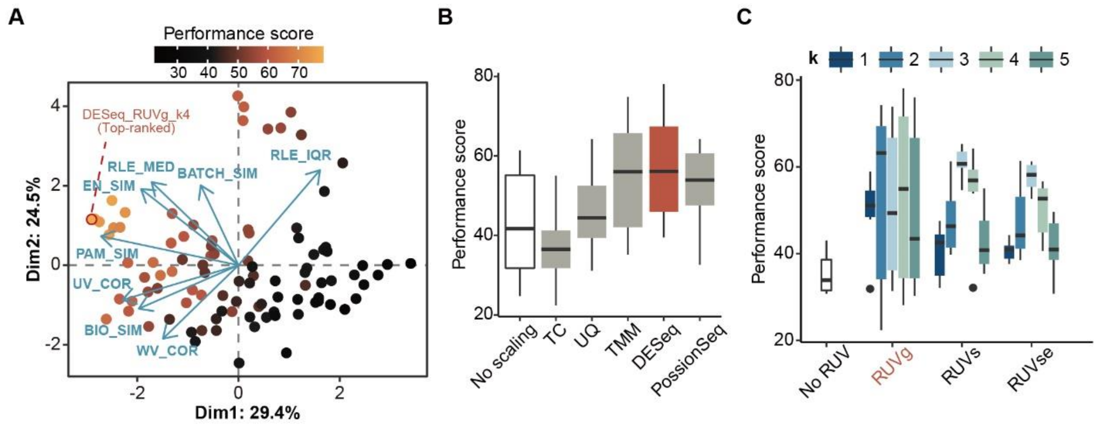

Figure S2 related to Figure 2: enONE normalization on the NAD-RNA-seq data. (A) enONE identified the top-ranked procedure (DESeq_RUVg_k4) from a total of 96 procedures. Each point corresponds to a normalization procedure and is colored by the performance score (mean of eight scone performance metric ranks). The blue arrows correspond to the PCA loadings for the performance metrics. The direction and length of a blue arrow can be interpreted as a measure of how much each metric contributed to the first two PCs. (B) Boxplot showing performance score, stratified by scaling procedures. (C) Boxplot showing performance score, stratified by regression-based procedures.

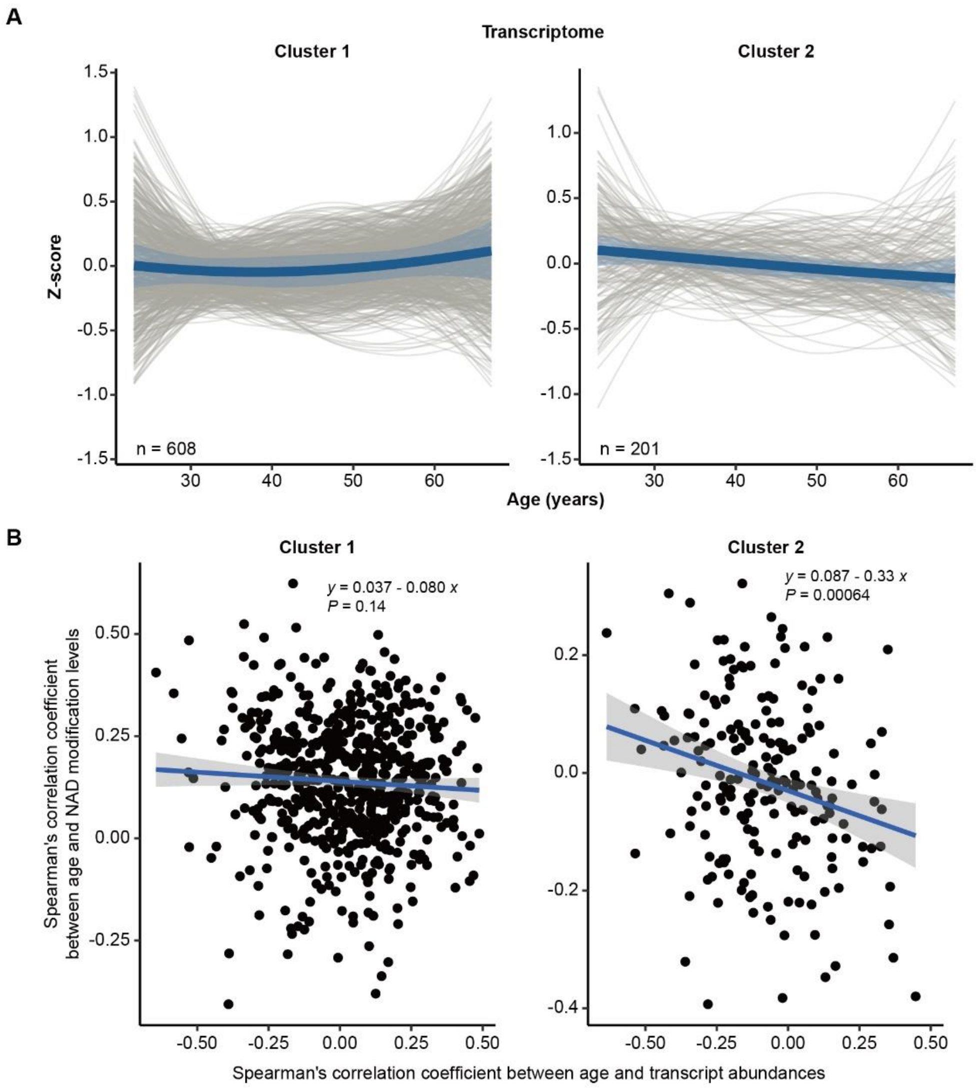

Figure S3 related to Figure 5: Assessment of Transcriptome of each cluster. (A) Gene expression trajectories of each cluster based on NAD capping dynamics. The solid line and shaded region represent the smoothed trajectory of each cluster and its 95% confidence intervals, respectively. (B) Correlation between age and NAD modification was not strongly associated with that between age and gene expression (*P*-values were assessed using *t* test). Each dot represents a gene.

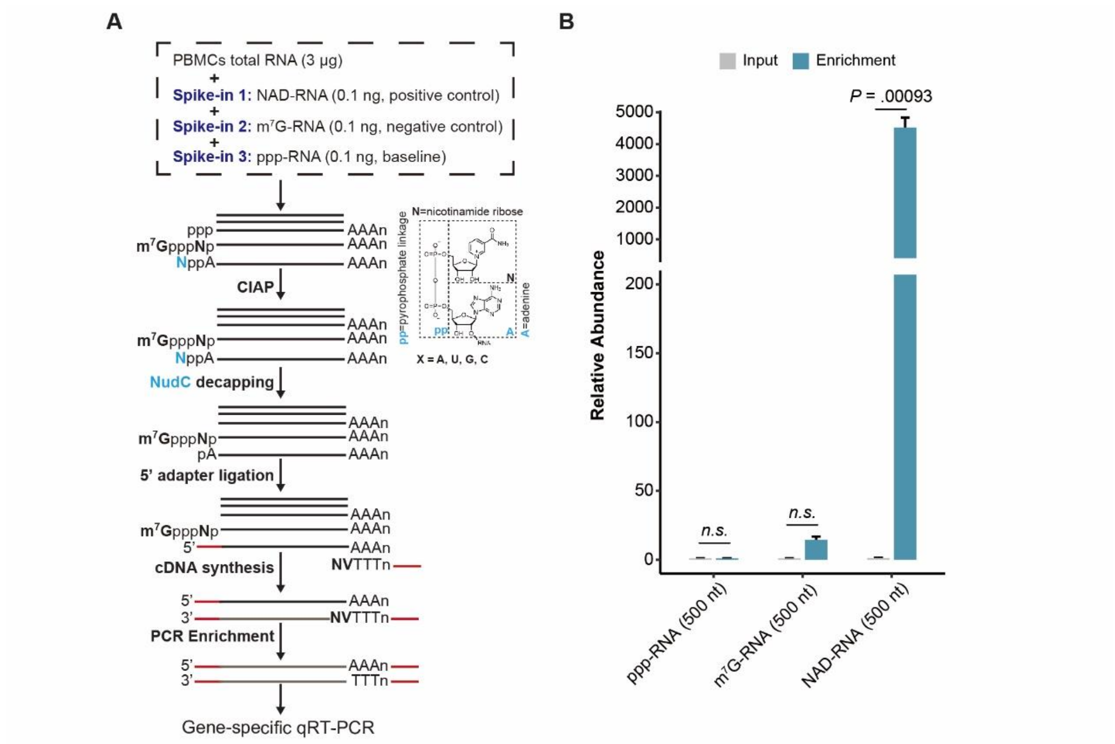

Figure S4 related to Figure 5: Validation of NAD-RNAs by modified CapZyme-Seq. (A) Diagram illustrating the workflow of modified CapZyme-Seq. (B) Control experiment of synthetic RNA confirms that spike-in NAD-RNA, but not ppp-RNA and m^{7}G-RNA, were significantly and selectively enriched. Data are mean ± s.e.m. (two-sided Student's *t* test, *n.s.* denotes not significant).

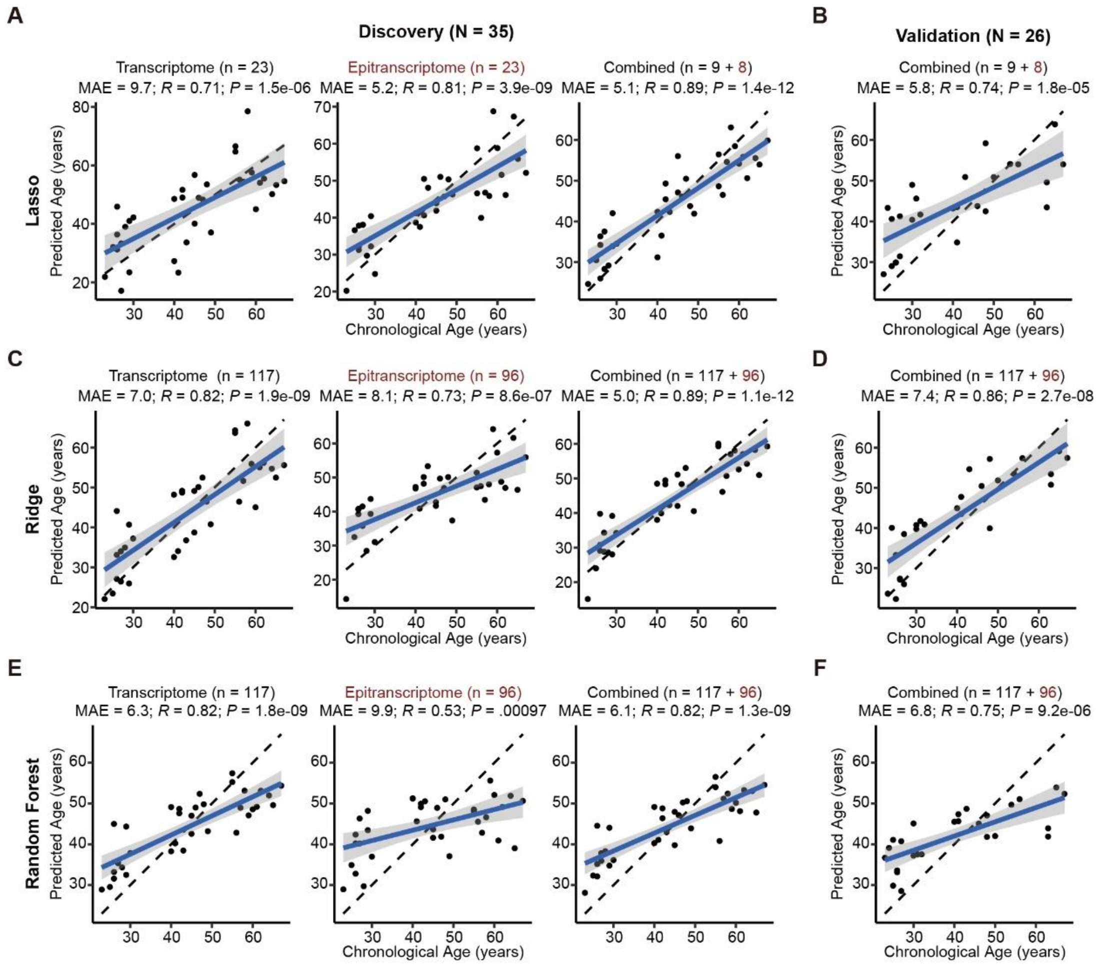

Figure S5 related to Figure 6: Age prediction models. Performance of age prediction models based on transcriptome (left), NAD-modified epitranscriptome (middle) and the combination of transcriptome and NAD-modified epitranscriptome (right) in the discovery cohort using lasso (A), ridge (C), and random forest regression (E). Performance of the combined age prediction model in the validation cohort based on model trained with lasso (B), ridge (D), and random forest regression (F). Epitranscriptomic features were colored in red.
